# Neuroinflammation in Central Nervous System Tumors

**DOI:** 10.3390/cells15171612

**Published:** 2026-09-04

**Authors:** Cristina Cueto-Ureña, María Jesús Ramírez-Expósito, José Manuel Martínez-Martos

**Affiliations:** Experimental and Clinical Physiopathology Research Group CTS-1039, Department of Health Sciences, School of Health Sciences, University of Jaén, E23071 Jaén, Spain; ccueto@ujaen.es (C.C.-U.); mramirez@ujaen.es (M.J.R.-E.)

**Keywords:** neuroinflammation, glioblastoma, tumor microenvironment, tumor-associated macrophages, cancer neuroscience, synaptic integration, immunosuppression, myeloid-derived suppressor cells, immunotherapy, clinical trials

## Abstract

**Highlights:**

**What are the main findings?**

- Neuroinflammation in CNS tumors, especially glioblastoma, is an active driver of tumor progression, rather than a passive bystander, through coordinated interactions among myeloid cells, astrocytes, lymphocytes, and tumor cells within the microenvironment.
- Tumor-promoting inflammation is sustained by immunosuppressive signaling, metabolic reprogramming, and neuron–glioma communication, with distinct patterns across IDH-wildtype gliomas, IDH-mutant gliomas, and brain metastases.

**What are the implications of the main findings?**

- Effective therapies will likely need to target several linked processes at once, including myeloid immunosuppression, metabolic adaptation, and bioelectric tumor-neural signaling, rather than relying on single-pathway approaches.
- Integrated biomarkers from imaging, liquid biopsy, and microenvironmental profiling may improve patient stratification and support the translation of emerging strategies such as CSF1R inhibition, immune checkpoint modulation, and next-generation cellular immunotherapy.

**Abstract:**

Neuroinflammation within the tumor microenvironment (TME) of central nervous system (CNS) neoplasms, particularly glioblastoma (GBM), is no longer viewed merely as a reactive phenomenon but rather as a major driver of gliomagenesis and malignant transformation. This process involves a shift from acute immune activation to a chronic, sterile state that reshapes the CNS borders and immune niches to favor tumor evasion. This narrative review provides a comprehensive mechanistically focused analysis of the mechanisms governing the inflammatory stroma in primary and metastatic brain neoplasms. It critically examines the ontogeny and transcriptomic profile of myeloid and glial populations, dismantling the binary M1/M2 polarization model in favor of a continuum of functional states determined by metabolic and oxygenation gradients. It also analyzes intracellular signaling cascades, the subversion of innate immunity sensors such as the cGAS-STING pathway, the epigenetic reprogramming of stromal cells, and the role of extracellular vesicles. The electrochemical integration of tumor cells into neuronal circuits via glutamatergic synapses and connexin 43 gap junction coupling is addressed in detail, defining the mitogenic impact of neuronal activity on the tumor. The inflammatory profiles of IDH-wildtype and IDH-mutant gliomas and of secondary brain metastases are contrasted. Finally, the correlates of functional neuroimaging, liquid biopsies, and resistance mechanisms to conventional therapies are analyzed, including the GIANT and SENIPERA clinical trials, CARv3-TEAM-E bivalent cellular immunotherapy preconditioned with the LDC + R regimen, and the accelerated approval of dordaviprone (Modeyso) in H3 K27M-mutant diffuse midline gliomas.

## 1. Introduction

The historical conceptualization of the central nervous system (CNS) as a compartment endowed with absolute immune privilege has been critically refuted by contemporary neuroimmunology [1]. The anatomical and physiological barriers of the brain parenchyma do not preclude highly dynamic immunological communication [2]. Beyond endogenous factors, emerging evidence from Mendelian randomization identifies environmental stressors, such as elevated circulating perfluorooctanoic acid (PFOA), as causal risk factors for glioblastoma (GBM) development, potentially mediated through the AGE-RAGE signaling axis and chronic neuroinflammation [3]. The discovery of functional lymphatic vessels in the dura mater meninges and the mapping of cerebrospinal fluid (CSF) flow and glymphatic clearance of solutes toward the deep cervical lymph nodes have demonstrated the existence of active and specialized immune surveillance under homeostatic conditions [2].

In the context of intracranial neoplasms, particularly in glioblastoma (GBM) and brain metastases (BrMs), this parenchymal immunity is profoundly co-opted by tumor cells to perpetuate a state of chronic, sterile, and intensely immunosuppressive neuroinflammation that promotes disease progression [4,5,6]. The inflammatory stroma in high-grade World Health Organization (WHO) gliomas constitutes a massive fraction, accounting for between 30% and 50% of the total tumor mass [7,8]. Far from executing cytotoxic programs oriented toward the elimination of mutated cells, immune and resident glial cells are phenotypically and molecularly reprogrammed through a dense exchange of chemotactic factors, immunomodulatory metabolites, and extracellular vesicles (EVs) [9].

The resulting inflammatory microenvironment suppresses T-cell-mediated adaptive immunity, accelerates aberrant neovascularization, alters cerebral endothelial tight junctions causing intractable vasogenic edema, and facilitates the diffuse invasion of glioma cells along white matter tracts and perivascular spaces [10]. A recently identified critical driver of progression is the axonal injury induced by the expansion of tumor cells into the white matter. This damage triggers an active axonal death program known as Wallerian degeneration, mediated by the enzyme SARM1, which intensifies neuroinflammation and tumor proliferation in a feedback loop [11]. Consequently, understanding neuroinflammation in neuro-oncology requires an integrative mechanistic approach, focused on deciphering the cellular ontogeny of stromal populations, the signal transduction cascades that control their transcriptome, and the complex metabolic and bioelectric interactions linking the tumor to the host neuronal and glial network [4,8,12]. Importantly, tumor-induced neuroinflammation shares core pathophysiological mechanisms with classical neurodegenerative diseases, including reactive astrogliosis, microglial-mediated synaptic loss, and Wallerian degeneration, bridging the fields of neuro-oncology and neurodegeneration [11,13].

## 2. Cellular Components of the Inflammatory Microenvironment

### 2.1. Tumor-Associated Macrophages

The dominant myeloid population within the malignant glioma tumor microenvironment collectively encompasses tumor-associated macrophages (TAMs), which present a well-defined ontogenic duality: the resident microglia of the brain parenchyma and bone marrow-derived macrophages (BMDMs) [4].

Microglia originate exclusively from uncommitted immature erythromyeloid progenitors of the primitive yolk sac during the early stages of embryonic hematopoiesis, migrating into the neuroepithelium before the physical establishment of the BBB and self-renewing locally throughout adult life under the guidance of trophic signals mediated by the CSF-1R receptor and its ligands CSF-1 and IL-34 [14,15]. In contrast, BMDMs originate from definitive hematopoietic stem cells of the adult bone marrow, circulate in the peripheral blood as classical inflammatory monocytes (Ly6C^high^ in mice), and actively cross the compromised blood-tumor barrier (BTB) in response to chemotactic signals secreted by the tumor [16].

Transcriptional signatures identified via scRNA-seq now distinguish resident microglia from bone marrow-derived macrophages (BMDMs) [16]. Crucially, brain immune evasion is driven by immuno-ageing processes; a critical threshold at approximately 57 years of age marks a significant decline in blood–brain barrier (BBB) integrity and an 85% increase in the extravasation of monocyte-derived macrophages. These cells adopt a suppressive phenotype regulated by the TREM2/TIM3 axis, which correlates directly with inferior survival outcomes in both GBM and neurodegenerative contexts [17]. Resident microglia maintain the constitutive expression of homeostatic markers such as TMEM119, P2RY12, Sall1, and CX3CR1, exhibiting a CD45low immunophenotypic profile [14]. However, this homeostatic signature is progressively lost during tumor-driven reprogramming, often involving the downregulation of P2RY12, which can be non-invasively monitored using anti-inflammatory-specific PET tracers like [^18^F]QTFT [18]. Furthermore, the myeloid landscape in recurrent glioblastoma (rGBM) is characterized by a significant expansion of SPP1+ immunosuppressive TAMs, which cooperate with hypoxic mesenchymal-like glioma stem cells to drive malignancy [14,19,20]. Conversely, BMDMs express extremely high levels of CD45 and integrin alpha 4 (CD49d), allowing their isolation under the CD45^high^CD11b^+^IBA1^+^ profile, accompanied by increased expression of legumain (LGMN) and the scavenger receptor CD163 [16].

Pathophysiologically, the cell lineage determines both the topographical distribution and the functional activation dynamics within the tumor [4,21].

Resident microglia predominate almost exclusively at the invasive tumor edge and in the peritumor zone adjacent to healthy brain tissue. They act as the first innate immune responder to oncogenesis but undergoes transcriptional remodeling induced by tumor factors that decreases its homeostatic markers (TMEM119, P2RY12) and activates genes involved in extracellular matrix remodeling to facilitate the diffuse infiltration of glioma cells [22].

**Figure 1:**
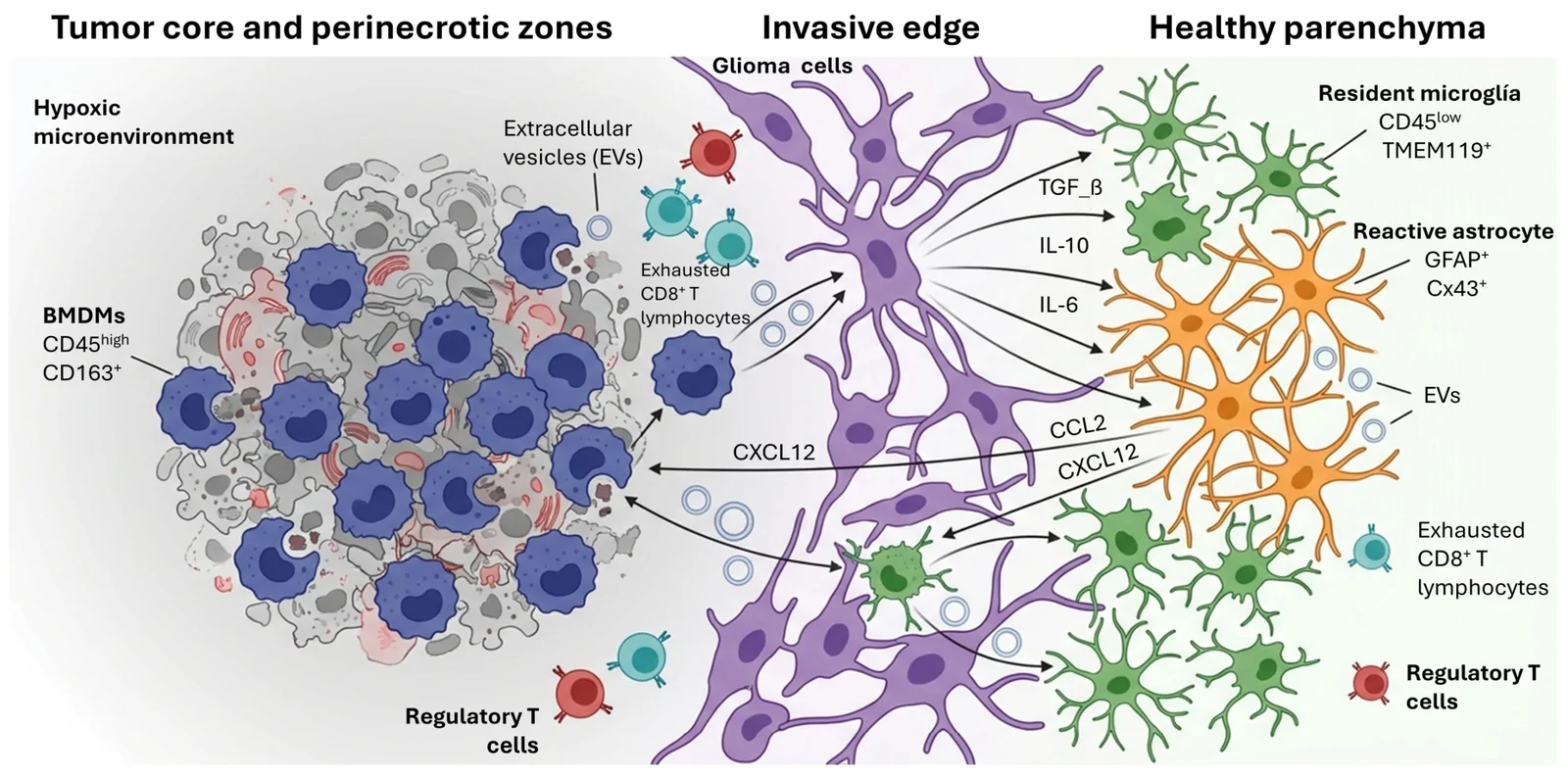
Cellular architecture of the glioblastoma immune microenvironment. Schematic representation of the topographical distribution of resident microglia (CD45^low^, TMEM119^+^), bone marrow-derived macrophages (CD45^high^, CD163^+^) in the tumor core and perinecrotic zones, reactive astrocytes (GFAP^+^, Cx43^+^) at the invasive edge, and scarce infiltrating exhausted CD8+ T lymphocytes and Tregs. Arrows indicate cytokine, chemokine, and extracellular vesicle-mediated interactions.

Regarding infiltrating BMDMs, they accumulate massively in the hypervascularized tumor core and in perinecrotic and hypoxic niches (Figure 1). Being recruited at more advanced stages, BMDMs are strongly reprogrammed by the metabolic and oxygen tensions of the TME, acquiring a transcriptome characterized by elevated levels of immunosuppressive factors (ARG1, IL10, TGFB1), proangiogenic mediators (VEGFA), and matrix metalloproteinases (MMP9, MMP2), thereby serving as major mediators of pathological angiogenesis and adaptive immune suppression [21,23]. Furthermore, microglia serve as the primary source of extracellular vesicle-derived miR21 during early oncogenesis, acting as a rapid-response element that precedes detectable tumor-cell-derived signaling [24]. The spatial organization of this response is further refined by S100A protein gradients, where S100A13 is specifically linked to microglial dysfunction and S100A11 modulates perivascular macrophage activity [25]. Additionally, the up-regulation of IL-10 in these glioma-associated microglia is mechanistically driven by the downregulation of the constitutive transcription factor USF-1, which normally represses IL-10 transcription [24,26]. The spatial architecture of this inflammation is further refined by S100A protein gradients: S100A11 localizes to vascular areas to modulate pericyte and macrophage function, while S100A13 is specifically linked to microglial dysfunction [25].

### 2.2. Tumor-Associated Astrocytes

Astrocytes, while initially protective, transition into a chronic detrimental state that facilitates blood–brain barrier (BBB) disruption and excitotoxicity [27]. A hallmark of astrocytic dysfunction in GBM is the loss of aquaporin-4 (AQP4) polarity. This depolarization uncouples the fluidic connectome, impairing glymphatic transport and metabolic buffering, which ultimately disrupts global neural network synchronization [28]. This state is exacerbated by solid stress from the expanding tumor mass, which activates AP-1-driven gene expression in glia, reinforcing a neurotoxic and proinflammatory feedback loop [29]. Such glymphatic failure is often accompanied by unilateral hippocampal atrophy, even in non-tumor inflammatory mimics [30].

In the tumor and peritumor parenchyma, TAAs increase the expression of glial fibrillary acidic protein (GFAP) and vimentin, remodeling their cytoskeleton toward a hypertrophic morphology [31]. TAAs secrete dense extracellular matrix components such as chondroitin sulfate proteoglycans and tenascin-C that alter tissue viscoelastic stiffness, sending mechanotransductive signals that stimulate glioma migration [31]. Likewise, TAAs establish direct contact with tumor cells through gap junctions composed of connexin 43 (Cx43) [32]. This functional syncytium allows the bidirectional transit of calcium ions (Ca^2+^) and cyclic adenosine monophosphate (cAMP) from tumor cells to astrocytes, activating in the latter transcriptional cascades that culminate in the release of pro-survival growth factors such as brain-derived neurotrophic factor (BDNF) and glial cell line-derived neurotrophic factor (GDNF), which rescue glioma cells from chemotherapy- and radiotherapy-induced oxidative stress [32]. Furthermore, TAAs promote the recruitment of additional TAMs through the production of the chemokines CCL2 and CXCL12, and constitutively secrete TGF-β and express PD-L1, directly participating in the paralysis of effector CD8+ T lymphocytes [33].

### 2.3. Myeloid-Derived Suppressor Cells and Neutrophils

The systemic and intratumor expansion of immature myeloid cells genetically blocked in their terminal differentiation process gives rise to myeloid-derived suppressor cells (MDSCs), which represent a key pillar in immunological evasion in glioma [34,35]. MDSCs are pathophysiologically classified into two main subpopulations; (1) Polymorphonuclear or Granulocytic MDSCs (PMN-MDSCs): Identified in humans as CD11b^+^CD14^−^CD15^+^CD33^+^ (Ly6G^+^Ly6C^low^ in rodent models) [34]. Their recruitment to the brain is mediated by the interaction of CXC motif chemokines (CXCL1, CXCL2, CXCL8/IL-8) with the myeloid receptor CXCR2. They exert T cell suppression mainly through the synthesis of reactive oxygen species (ROS) and peroxynitrite, inducing physical nitration of T cell receptor (TCR) residues and preventing the recognition of the peptide antigen bound to MHC [35]. (2) Monocytic MDSCs (M-MDSCs): Immunophenotypically defined as CD11b^+^CD14^+^HLA^−^DR^−/low^CD15^−^ (Ly6C^high^Ly6G^−^ in mouse) [34]. These cells traffic in response to the chemokine CCL2 gradient and express elevated levels of arginase-1 (ARG1) and inducible nitric oxide synthase (iNOS/NOS2). The accelerated enzymatic consumption of extracellular L-arginine by arginase-1 deprives effector T lymphocytes of this essential amino acid, causing the transcriptional destabilization of the TCR CD3ζ signal transducing chain and halting adaptive cell proliferation in the G0-G1 phase of the cell cycle [35].

In parallel, tumor-associated neutrophils (TANs) infiltrate high-grade brain neoplasms, particularly at necrosis margins and areas of aberrant neovascularization, attracted by CXCL8 and IL-1β gradients. Under the influence of tumor TGF-β, these neutrophils acquire a pro-tumor polarization (N2), secreting metalloproteinases (MMP-9) and neutrophilic elastase to facilitate cell invasion, and extruding neutrophil extracellular traps (NETs) [34]. NETs, three-dimensional networks of decondensed DNA and cationic granular proteins, act as a physical barrier that selectively conceals tumor antigens from immune surveillance, while interacting with receptors on glioma stem cells (GSCs) to induce mitochondrial biogenesis, metabolic plasticity, and selective resistance to chemotherapy and radiotherapy [34].

### 2.4. Lymphoid Infiltration and T Cell Exhaustion

The adaptive immune compartment in glioblastoma and other malignant brain tumors is notably scarce and ineffective, classifying these pathologies under the phenotype of “immunologically cold” or “desert” tumors [36]. The scarce tumor-infiltrating lymphocytes (TILs) that manage to access the parenchyma face a hostile biochemical environment that induces their terminal functional exhaustion [36].

Exhausted effector CD8+ T cells exhibit the persistent and sustained co-expression of multiple inhibitory surface immune checkpoint receptors, including programmed death receptor 1 (PD-1), cytotoxic T lymphocyte-associated antigen 4 (CTLA-4), T cell immunoglobulin and mucin domain 3 (TIM-3), lymphocyte activation gene 3 (LAG-3), and T cell immunoreceptor with Ig and ITIM domains (TIGIT) [36]. The PD-L1 ligand, overexpressed on the surface of glioma cells and surrounding TAMs due to tissue hypoxia and tonic IFN-γ signaling, interacts with the PD-1 receptor on lymphocytes, triggering the recruitment of the phosphatase SHP-2. The phosphatase dephosphorylates the tyrosine residues of the TCR CD3ζ chain and the CD28 costimulatory molecule, inhibiting downstream signaling of the PI3K and Akt kinases, which blocks aerobic effector glycolysis and abolishes the production of the effector interleukins IL-2, TNF-α, and IFN-γ [36].

This adaptive cytotoxic paralysis is consolidated by the recruitment and selective retention of regulatory T lymphocytes (CD4^+^CD25^+^FoxP3^+^ Tregs), attracted to the tumor parenchyma through myeloid and tumor secretion of the chemokine CCL22, a ligand for the chemokine receptor CCR4 enriched in Tregs [37]. Tregs neutralize local effector immunity by actively consuming available extracellular IL-2 through their constitutive expression of the high-affinity IL-2 receptor alpha subunit (CD25), constitutively secreting IL-10 and TGF-β, and expressing the surface ectoenzymes CD39 and CD73 [37,38]. These nucleotidases sequentially degrade proinflammatory extracellular ATP released by the tumor into adenosine, which couples to the A2A metabotropic receptors expressed on effector T cells. A2A receptor signaling increases intracellular cyclic AMP (cAMP) concentration and induces protein kinase A (PKA) activation, blocking the transcription of proinflammatory cytokines and forcing a state of profound local immunological tolerance [38] (Figure 1).

### 2.5. Axonal Injury and Complement Signaling

A critical and previously underappreciated driver of GBM progression is tumor-induced axonal injury in the white matter. Mechanistically, early tumor expansion triggers Wallerian degeneration via the SARM1 enzyme, which establishes a proinflammatory feedforward loop that enhances tumor proliferation [11]. The C5a/C5aR1 axis is a central orchestrator of tumor-macrophage symbiosis, where GSC-derived C5a promotes TAM infiltration via the p-AKT T308 pathway [39]. Beyond tumor growth, this axis mediates radiation-induced cognitive decline (RICD); pharmacological inhibition of C5aR1 with brain-penetrant antagonists, such as PMX205, preserves synaptic integrity and reverses neurocognitive deficits without compromising the tumor-killing efficacy of radiotherapy [40].

The principal cellular components of the CNS tumor immune microenvironment, together with their ontogeny, markers, and major pathophysiological roles, are summarized in Table 1.

### 2.6. PANoptosis as a Cell Death Mechanism

Beyond apoptosis and necrosis, CNS tumors and neuroinflammatory disorders are increasingly defined by PANoptosis, a coordinated programmed cell death pathway integrating features of apoptosis, necroptosis, and pyroptosis [41]. Regulated by the PANoptosome complex, this pathway serves a dual role: it can eliminate malignant cells but often exacerbates chronic neuroinflammation via a functional transition from acute VDAC1-mediated neuronal stress to chronic TAK1-dependent glial activation [42]. Targeting the molecular nodes of the PANoptosome represents a novel frontier for balancing antitumor efficacy with the preservation of the neural parenchyma [42].

## 3. Signalling Pathways and Immune Evasion

### 3.1. Chemotactic Pathways

The massive infiltration and precise positioning of stromal myeloid populations within the glioma tumor architecture are actively regulated by redundant networks of soluble chemokines secreted by both tumor cells and reactive astrocytes at the periphery [4]. The primary chemotactic recruitment is governed by the chemotactic axes CCL2-CCR2, CXCL12-CXCR4, and CX3CL1-CX3CR1 [43,44,45].

Regarding the CCL2-CCR2 axis, the transmigration of inflammatory monocytes through the disrupted BTB is dictated by this axis. Glioma cells actively transcribe CCL2 (MCP-1) due to tonic NF-κB- and STAT3-mediated signaling [43]. The binding of CCL2 to its cognate receptor CCR2 on peripheral classical monocytes induces a G protein-mediated phosphorylation cascade, mobilizing intracellular calcium and triggering cytoskeleton polymerization programs that lead to endothelial adhesion and parenchymal infiltration [43].

Regarding the CXCL12-CXCR4/CXCR7 Axis, intratumor hypoxia stimulates CXCL12 (SDF-1) transcription in a HIF-1α-dependent manner [44]. CXCL12 acts as a potent chemoattractant for TAMs and MDSCs expressing CXCR4, sequestering them within perinecrotic niches, where they actively cooperate in inducing neovascularization and maintaining GSC pluripotency [19].

Finally, the CX3CL1-CX3CR1 axis is essential for homeostasis and physiological synapse pruning mediated by microglia, and it is proteolytically subverted by glioma metalloproteinases (ADAM10 and ADAM17), releasing a soluble form of fractalkine (CX3CL1) that chemotactically sequesters resident microglia toward the adjacent tumor periphery for subsequent transcriptional reprogramming [33,45].

### 3.2. Immunosuppressive Cytokines

Once TAMs and other innate immune cells are localized within the tumor niches, their operational characteristics are profoundly reprogrammed by a cocktail of immunosuppressive cytokines dominated by transforming growth factor beta (TGF-β), interleukin-10 (IL-10), and interleukin-6 (IL-6) [5].

TGF-β (of which the glioma actively secretes the TGF-β1 and TGF-β2 isoforms) binds to its heterodimeric type I/II transmembrane receptor (TGFBR1/2) endowed with serine/threonine kinase activity on immune cells. This ligation induces the selective phosphorylation of the cytoplasmic transducing proteins Smad2 and Smad3, which physically associate with Smad4 and translocate to the nucleus. The resulting multiprotein complex directly represses the transcription of genes encoding effector proinflammatory cytokines such as IL-12, while coordinately inducing genes involved in stromal remodeling and in the synthesis of proangiogenic factors such as VEGF, thereby promoting the suppressive phenotypic transition of TAMs [5].

IL-10, secreted by both tumor cells and reprogrammed TAMs, signals through the IL-10R1/2 complex to activate the JAK1/Tyk2/STAT3 axis. This pathway is further amplified by the loss of upstream stimulating factor 1 (USF-1) in microglia, which leads to sustained IL-10 mRNA expression and subsequent MHC class II downregulation, effectively shielding the tumor from adaptive surveillance [26,46].

IL-6 behaves as a pleiotropic cytokine of high pathophysiological relevance, whose elevated levels in the parenchyma and CSF correlate directly with tumor grade and decreased survival [45]. Upon binding to the membrane or soluble IL-6 receptor (IL-6R), IL-6 induces the homodimerization of the gp130 signal transducer, triggering the activation of the JAK/STAT3 pathway in both tumor and stromal cells. In tumor cells, this signaling promotes autocrine survival, mitogenic self-renewal of glioma stem cells (GSCs), and chemotherapeutic resistance; in adjacent innate immune populations, IL-6 paracrinely perpetuates the suppressive polarization and recruitment of MDSCs, structuring a proinflammatory but intensely immunotolerant feedback loop [45].

### 3.3. NF-κB and STAT3 Signalling

At the convergence point of the soluble cytokine and metabolic signals that pervade the malignant glioma TME, there are two constitutively active transcriptional hubs: STAT3 and the nuclear factor kappa B (NF-κB) pathway [16,47].

- The transducer STAT3 is persistently phosphorylated at its tyrosine 705 residue (pY705-STAT3) in both tumor cells and TAMs, TAAs, and MDSCs, stimulated by the tonic secretion of IL-6, IL-10, and basic fibroblast growth factor (bFGF). After its phosphorylation, STAT3 homodimerizes and translocates to the nuclear compartment, where it binds to specific DNA response elements to activate the transcription of immunosuppressive and angiogenic effector genes such as IL10, TGFB, VEGFA, and PDL1, while repressing the transcription of the IL-12 p40 subunit and MHC-II molecules. Consequently, STAT3 operates as an intracellular molecular switch that blocks the development of a protective Th1-type immune response, driving both tumor-intrinsic stemness and stroma-mediated immune tolerance [16,39,46].

The activation of the canonical NF-κB pathway, dominantly mediated by the p50/p65 heterodimer, is stimulated by stromal proinflammatory factors and DAMPs released from foci of intratumor necrosis, including TLR2/4 ligands and TNF-α [47]. This signaling activates the IKK kinase complex, causing the phosphorylation and subsequent proteasome-mediated degradation of the inhibitory protein IκBα, releasing the p50/p65 dimer for nuclear entry. In glioma tumor cells, especially in those belonging to the mesenchymal transcriptomic subtype, NF-κB drives the massive transcription of pro-tumor cytokines (IL-6, IL-8), proangiogenic factors (VEGF), metalloproteinases, and anti-apoptotic genes (Bcl-xL, survivin), directly correlating with radioresistance and accelerated tumor progression [47].

NF-κB and STAT3 do not operate in isolated compartments; extensive cross-talk has been characterized in which NF-κB-dependent cytokines (such as IL-6) stimulate sustained STAT3 activation, and in turn, STAT3-dependent molecular marks prolong NF-κB retention in the cell nucleus. The cooperative coupling of both factors to contiguous promoter regulatory regions of specific genes perpetuates the tonic transcription of pro-tumor factors, blocking the resolution of the neuroinflammatory response and consolidating therapeutic resistance [45]. This cross-talk is further amplified by an IL-1β-PGE2/TNFα-EP4-C/EBPβ feedback loop, where TAMs secrete IL-1β to induce tumor-derived PGE2 and TNFα, which in turn activate C/EBPβ in macrophages to sustain IL-1β transcription [45]. Additionally, the purinergic P2X7 receptor (P2X7R) has been identified as a key driver of this multidirectional neuroinflammatory milieu, linking innate immunity markers (CD68, S100A9) with tumor markers like MMP9 and VEGFB [48].

### 3.4. cGAS-STING and the NLRP3 Inflammasome

The detection of free cytoplasmic DNA through the enzymatic cyclic GMP-AMP synthase (cGAS)-stimulator of interferon genes (STING) pathway represents a fundamental innate immune barrier [49]. In glioma, chromosomal instability (CIN) frequently leads to the formation of genomic micronuclei, which are prone to nuclear envelope rupture, releasing double-stranded DNA (dsDNA) into the cytosol as a cell-intrinsic danger signal [49]. Normally, cytosolic dsDNA activates cGAS to catalyze cGAMP synthesis, triggering STING activation and the subsequent expression of type I interferons (IFNs) and proinflammatory cytokines. However, glioma cells frequently undergo epigenetic silencing of cGAS and STING expression to suppress this pathway and evade immune surveillance [41]. To overcome this evasion, STING agonists are being actively evaluated in clinical trials to reactivate local type I IFN signaling and reprogram the immunosuppressive tumor microenvironment [41,49]. Simultaneously, as demonstrated in brain metastatic breast and lung carcinoma cells, tumor cells can channel the cyclic dinucleotide cGAMP, generated residually by their own cGAS machinery, directly into the cytoplasm of parenchymal astrocytes through Cx43 gap junctions [50]. In the recipient astrocytes, cGAMP activates host STING signaling. Pathophysiologically, this chronic stromal STING activation does not lead to a purging immune response but induces the expression of pro-tumor soluble cytokines, including TNF-α and IL-6. These astrocytic mediators paracrinely feedback to glioma cells, stimulating their own NF-κB- and STAT3-controlled cell survival pathways and promoting active tumor progression [50].

**Figure 2:**
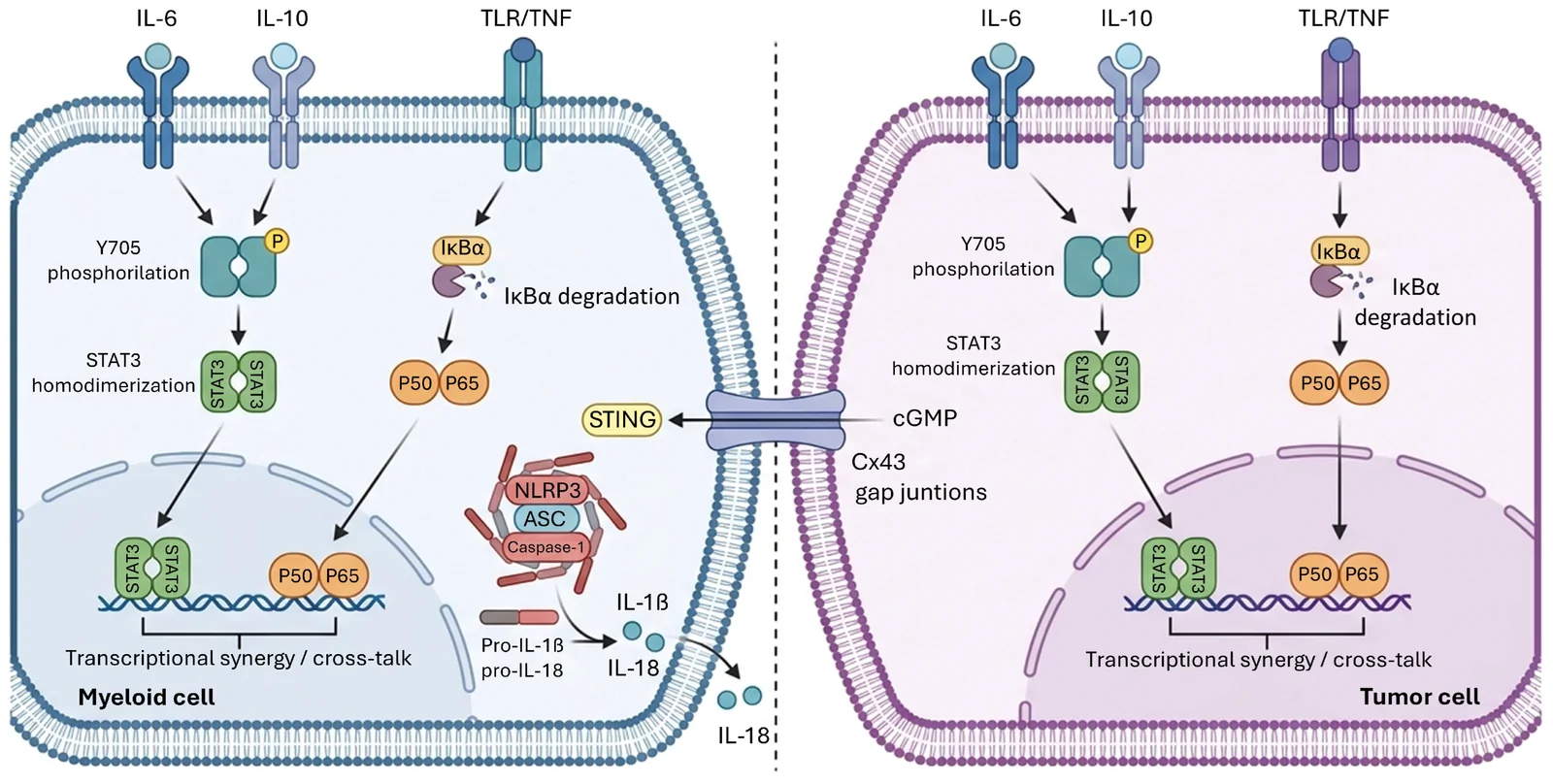
Intracellular signaling pathways in myeloid and tumor cells within the glioma TME. Diagram depicting STAT3 activation (Y705 phosphorylation) by IL-6 and IL-10; the canonical NF-κB pathway (IκBα degradation, nuclear translocation of p50/p65); the cGAS-STING pathway and its subversion through cGAMP transfer via Cx43 gap junctions; and the NLRP3 inflammasome with caspase-1 activation and IL-1β/IL-18 maturation. Convergence nodes between STAT3 and NF-κB are indicated.

In parallel, the myeloid NLRP3 inflammasome is intensely stimulated within TAMs in response to the release of intratumor DAMPs, such as accumulated extracellular ATP and mitochondrial reactive oxygen species in areas of active necrosis [41]. The multiprotein nucleation of NLRP3 and the recruitment of the adaptor protein ASC promote the proteolytic activation of caspase-1, catalyzing the maturation of the interleukins IL-1β and IL-18. Far from inducing immunogenicity, the constant secretion of IL-1β into the TME locally amplifies the NF-κB and STAT3 pathways in tumor cells, stimulates endothelial VEGF expression promoting angiogenesis, and chemotactically accelerates the arrival of additional suppressive MDSCs, consolidating tumor progression [41] (Figure 2).

## 4. Immunometabolism and Epigenetics

### 4.1. Amino Acid Catabolism

The selective depletion of essential amino acids within the glioma extracellular space exerts strict control over the operational dynamics of immune cells [51]. The rate-limiting clearance of L-tryptophan is catalyzed by the overexpressed enzymes indoleamine 2,3-dioxygenase 1 (IDO1) and tryptophan 2,3-dioxygenase (TDO2) in tumor cells and polarized TAMs [51]. This metabolic catabolism exerts a dual immunosuppressive blockade:

The accelerated depletion of tryptophan deprives infiltrating cytotoxic T lymphocytes of this amino acid, which is immediately sensed through the accumulation of uncharged tRNAs. This metabolic signaling allosterically activates the protein kinase GCN2 (general control nonderepressible 2) in the lymphocyte cytoplasm. The activated GCN2 kinase initiates a phosphorylation cascade that broadly blocks the protein translation initiation machinery of the T cell, forcing cell cycle arrest, adaptive anergy, and T cell apoptosis [51].

Simultaneously, the resulting accumulation of extracellular L-kynurenine is actively imported into the cytoplasm of the TAMs themselves and effector T cells, where it operates as a high-affinity endogenous ligand of the aryl hydrocarbon receptor (AhR), a ligand-dependent cytoplasmic transcription factor [51]. After kynurenine binding, AhR translocates to the nucleus, where it dimerizes with the AhR nuclear translocator (ARNT) and directs a robust tolerogenic transcriptional program. This program directly promotes the differentiation of naive CD4 lymphocytes toward the Treg suppressive lineage through the transactivation of FOXP3, induces the up-regulation of the immunosuppressive ectoenzymes CD39 and CD73 on TAMs, and stimulates the constitutive expression of PD-L1 on the myeloid surface, comprehensively inactivating cytotoxic immunity [51]. Despite the high expression of indoleamine-2,3-dioxygenase-1 (IDO1) in activated endothelial cells (ECs) within the glioma vascular niche, cell-specific ablation models suggest that endothelial IDO1 is not the dominant regulator of CNS antitumor immunity, pointing towards tumor-cell- and TAM-derived IDO1/TDO2 as the primary drivers of tryptophan-mediated immunosuppression [51].

### 4.2. Glycolysis, Lactate, and Lipid Reprogramming

Glioma tumor cells constitutively execute a high rate of aerobic glycolysis, characterized by accelerated glucose consumption and the consequent secretion of lactic acid into the interstitial space through monocarboxylate transporters (MCT1 and MCT4) [52]. This extracellular accumulation of lactate and the consequent acidification of the myeloid cellular TME (pH ~6.5–6.8) operate as critical biochemical signals for macrophage and microglia populations [52].

TAMs actively import lactate from the microenvironment via MCT1, which increases cytoplasmic pyruvate concentrations and disrupts the proteasomal degradation of the transcription factor HIF-1α independently of actual extracellular oxygen tensions. The cytosolic stabilization of HIF-1α in TAMs, together with lactate-mediated STAT6 signaling pathway activation, directly represses the promoters of proinflammatory myeloid genes and robustly activates the transcription of ARG1, VEGFA, and CD206/MRC1, locking macrophages into an intensely suppressive and tissue-remodeling phenotype [52]. Recent evidence also identifies gender-specific metabolic control: androgen loss (via castration) accelerates intracranial tumor growth by hyperactivating the HPA axis through increased IL-1β and TNF signaling, which enhances inflammasome activation in microglia [23]. This highlights an organ-specific regulation of antitumor immunity driven by neuroendocrine-inflammatory cross-talk [23].

In parallel, a reprogramming in the lipid metabolism of infiltrating GBM TAMs has been characterized. The nutrient-deprived TME induces macrophages to perform a metabolic switch toward fatty acid oxidation (FAO) as a primary energy source. TAMs sustainably increase the uptake of lipids and lipid debris from apoptotic glioma cells through the scavenger receptors CD36 and triggering receptor expressed on myeloid cells 2 (TREM2). The resulting accumulation of intracellular lipid droplets and cholesterol esters disrupts the phagosome assembly machinery, abolishing the capacity for efficient phagocytosis, while acting as an intracellular stimulus for the tonic secretion of immunotolerant cytokines [16,17,52].

### 4.3. Epigenetic Regulation of Myeloid Tolerance

The states of immunological tolerance and the plasticity of myeloid cells in high-grade glioma depend not only on constant stimulation by extracellular ligands but are epigenetically consolidated [5]. TAMs exposed to the tumor microenvironment exhibit an altered chromatin landscape, characterized by the hypermethylation of the promoters of genes involved in cytotoxic immunity and antigen presentation (such as IL12, TNF and MHC class II loci), mediated by sustained DNA methyltransferase (DNMT) activity [5].

Simultaneously, histone deacetylases (HDACs, especially HDAC1, HDAC2, and HDAC6) are overexpressed in intratumor TAMs, deacetylating the lysine residues of histone tails at the promoter loci of costimulatory molecules, forcing a closed chromatin conformation (heterochromatin) that physically prevents transcription factors from accessing these proinflammatory genes. Conversely, the loci of immunosuppressive genes such as IL10 and ARG1 retain persistently open chromatin marks (such as trimethylation of histone 3 lysine 4, H3K4me3) [5].

This epigenetic imprint is reinforced by the transfer of non-coding RNAs via extracellular vesicles (EVs) released by tumor cells. Tumor EVs transport specific microRNAs (such as miR-21, miR-451, and miR-124) and long non-coding RNAs (such as H19 and NEAT1) directly to the myeloid cytoplasm. In the myeloid recipient, these RNAs post-transcriptionally suppress key regulatory targets of the NF-κB and STAT3 pathways, forcing a phenotype shift independently of the presence of extracellular soluble gradients [8,9,53]. In this niche, glioma stem cells (GSCs) preferentially secrete the complement component C5a, which acts on the C5aR1 receptor of TAMs. This signaling activates the p-STAT3-cMyc-PD-L1 axis in GSCs to promote self-renewal and recruits immunosuppressive macrophages through the C5aR1-p-AKT T308 axis [39].

### 4.4. The Microbiota–Gut–Brain-Tumor Axis

Systemic dysbiosis is now recognized as a potent modulator of the GBM microenvironment [54]. Glioma progression is consistently associated with a reduced Firmicutes:Bacteroidetes ratio and the depletion of neuroprotective short-chain fatty acids (SCFAs) [55]. This systemic inflammatory state impairs antitumor T-cell responses through antigenic mimicry and promotes angiogenesis, suggesting that microbiome-targeted interventions, including probiotics or SCFA supplementation, could serve as vital adjuncts to immune checkpoint therapy [55].

## 5. Synaptic Integration and Cancer Neuroscience

### 5.1. Neuron–Glioma Synapses

The emergence of “cancer neuroscience” has reconfigured the biological understanding of malignant gliomas, demonstrating that glioma cells do not simply physically invade the neural tissue but actively integrate into neuronal circuits through the establishment of genuine electrochemical synapses [12,56,57,58]. Glioma cells, including high-grade astrocytomas and diffuse midline gliomas, express specific structural proteins of the postsynaptic scaffolding, including Ca^2+^-permeable AMPA-type glutamate receptors, formed by combinations of GluA1–4 subunits [56,58].

These neuron–glioma synapses are fully functional. The presynaptic action potential evoked in cortical glutamatergic neurons causes the release of glutamate into the synaptic cleft, which binds to AMPA receptors on glioma cells, inducing rapid excitatory postsynaptic currents (EPSCs) lasting 4 to 5 milliseconds [56,57]. This tumor membrane depolarization triggers a massive influx of intracellular calcium that is amplified throughout the tumor because of intercellular electrical coupling caused by connexin 43 gap junctions in the tumor microtubules (TMs), propagating the signal to distant cells not synaptically connected [56,57].

This Ca^2+^ influx and oscillation activates intracellular mitogenic cascades, primarily the MAPK/ERK and PI3K-Akt-mTOR pathways, directly driving cell proliferation, tumorigenesis, and invasive cell invasion [56,58]. Membrane patch-clamp studies in fresh human brain slices with infiltrating glioma have revealed that the biophysical properties of cortical pyramidal neurons vary according to the histologic grade of the tumor [12]. Neurons in the cortex infiltrated by high-grade glioma exhibit a significantly higher state of intrinsic hyperexcitability compared to low-grade gliomas [12]. In parallel, glioma cells in high-grade tumors exhibit more prolonged and sustained synaptic responses [12]. This hyperexcitability of pyramidal circuits generates accelerated neuron–glioma network activity that directly feeds back and accelerates tumor cell mitosis [12].

The maintenance and plasticity of these synaptic connections are retrogradely regulated by neuronal activity. Beyond AMPA receptors, group II metabotropic glutamate receptors, specifically mGlu3, have emerged as critical targets; pharmacological blockade of mGlu3 receptors significantly reduces the incidence of gliomas and lowers Iba-1+ cell density, suggesting a reduction in the pro-tumorigenic neuroinflammatory infiltrate [59]. Neuronal depolarization induces the proteolytic cleavage and secretion of the synaptic adhesion molecule neuroligin-3 (NLGN3) by the metalloproteinase ADAM10 in the cerebral cortex [56,58]. Soluble NLGN3 binds to its receptor on the tumor cell, promoting the transcription of genes associated with synaptogenesis (including AMPA receptors and connexin subunits) and establishing a feedforward loop that wires the tumor into the neural network [56,58].

### 5.2. GABAergic Depolarization in Diffuse Midline Gliomas

A molecular aspect of particular importance in the pathophysiology of diffuse midline gliomas (DMGs) is the alteration in the intracellular chloride gradient, which subverts the function of the inhibitory neurotransmitter GABA [56,58]. Under physiological mature neuronal conditions, the binding of GABA to its ionotropic type A receptor (GABA_A) promotes a net influx of chloride ions (Cl-), inducing hyperpolarization that silences electrical excitability.

However, DMG cells present a constitutive overexpression of the sodium-potassium-chloride cotransporter 1 (NKCC1) together with a marked decrease or functional deletion of the chloride-potassium cotransporter 2 (KCC2) [56,58]. This results in abnormally elevated intracellular chloride concentrations: [Cl^−^]_i_ > [Cl^−^]_eq_, where the equilibrium potential for chloride (E_Cl) is depolarized with respect to the resting membrane potential.

**Figure 3:**
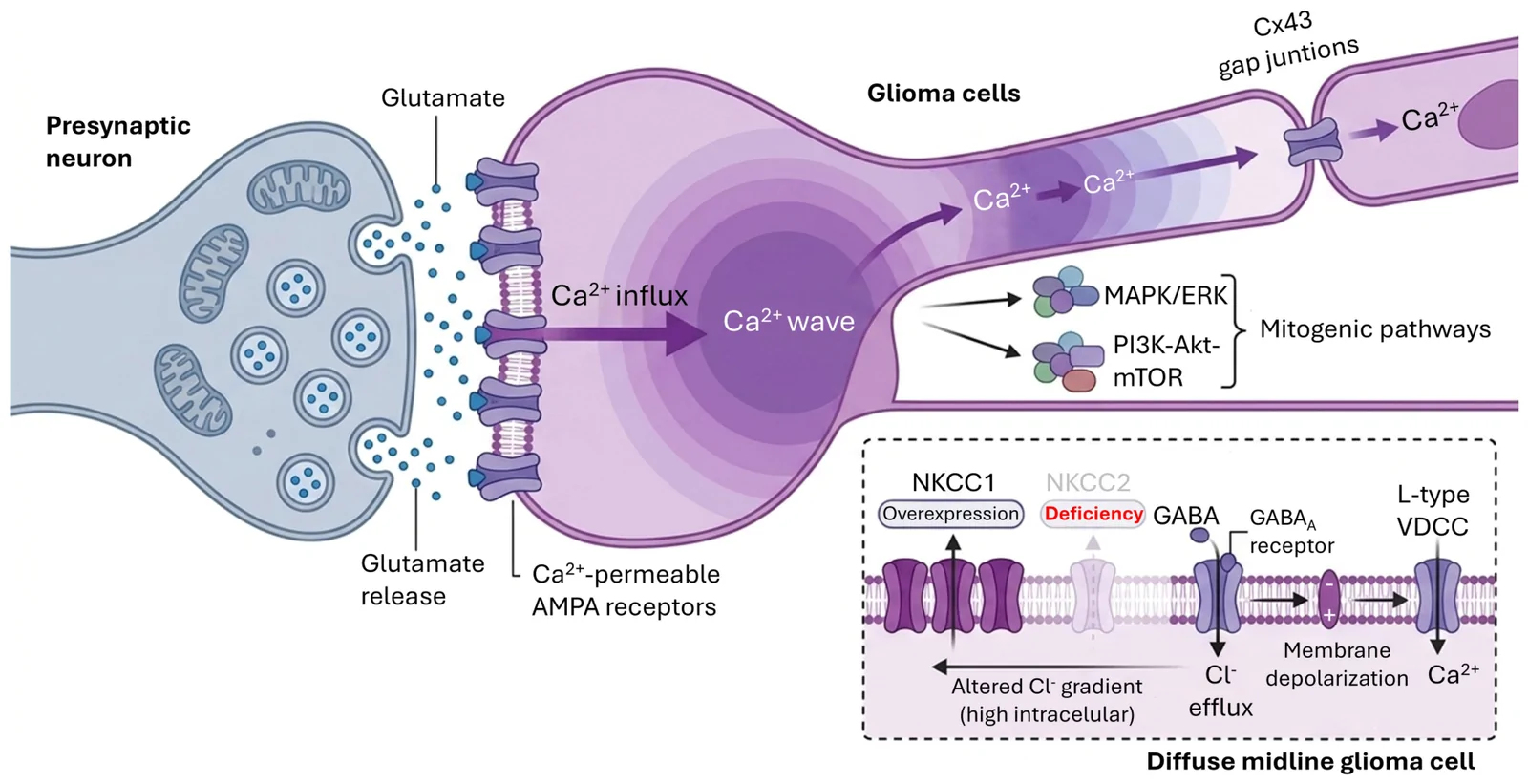
Neuron–glioma glutamatergic synapse and bioelectric integration. Glutamate is released from the presynaptic neuronal terminal, binds to calcium-permeable AMPA receptors on the glioma cell membrane, causes Ca^2+^ influx, propagates signals through Cx43 gap junctions between tumor cells, and activates MAPK/ERK and PI3K-Akt-mTOR mitogenic pathways. Inset: Altered Cl^−^ gradient in diffuse midline gliomas (DMG) with NKCC1 overexpression and KCC2 deficiency, resulting in GABAergic depolarization.

When GABA released by host presynaptic interneurons couples to the membrane receptors of DMG glioma cells, the electrochemical gradient pushes chloride toward the extracellular space, actively depolarizing the tumor membrane. This depolarization opens L-type voltage-dependent calcium channels (VDCCs), promoting the cyclic entry of Ca2+ that synergistically stimulates the mitogenic transcriptome and favors proliferation, converting a physiological inhibitory neurotransmitter into a bioelectric engine of tumor progression [56,58] (Figure 3).

## 6. Microenvironment by Tumor Type

### 6.1. IDH-Wildtype and IDH-Mutant Gliomas

The cellular architecture and soluble cytokine networks that constitute the neuroinflammatory stromal microenvironment of glioma are directly determined by the intrinsic tumorigenic genomic signatures, establishing a clear pathophysiological division between IDH-wildtype neoplasms and those possessing heterozygous mutations in the isocitrate dehydrogenase 1 or 2 genes (IDH1/2) [60].

In gliomas with IDH mutation (typical of low-grade astrocytomas and oligodendrogliomas), the mutated enzyme acquires a neomorphic activity that catalyzes the conversion of α-ketoglutarate into the oncometabolite D-2-hydroxyglutarate (2-HG) [60]. 2-HG accumulates intracellularly and is massively secreted into the parenchymal extracellular space [16]. Free 2-HG acts as a competitive inhibitor of α-ketoglutarate, blocking α-ketoglutarate-dependent dioxygenases, including histone demethylases and TET family DNA demethylases, consolidating the glioma CpG island methylator phenotype (G-CIMP) [61]. This hypermethylation transcriptionally represses the promoters of critical inflammatory chemokine genes such as CCL2, CXCL2, and CXCL10, mechanically preventing the recruitment and chemotaxis of circulating TAMs and MDSCs to the brain [16].

**Figure 4:**
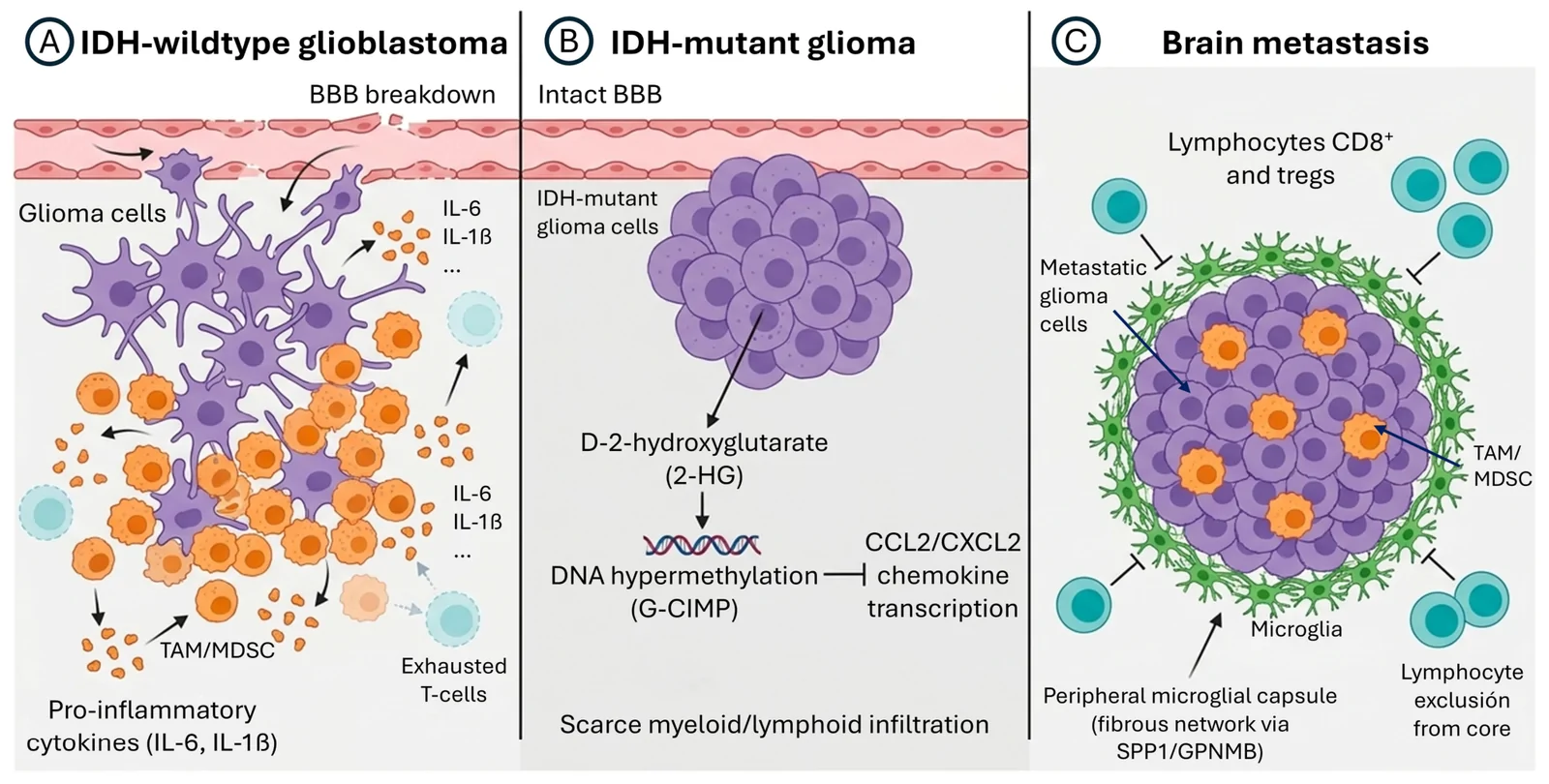
Comparative immune microenvironments according to tumor etiology. (**A**) IDH-wildtype glioblastoma: dense TAM and MDSC infiltration, high proinflammatory cytokine secretion, extensive BBB disruption, and scarce lymphoid infiltration. (**B**) IDH-mutant glioma: 2-HG production, epigenetic chemokine silencing, scarce myeloid infiltration, and immunologically “cold” microenvironment. (**C**) Brain metastasis: Nodular mass, peripheral microglial capsule, and lymphocyte exclusion from the tumor core.

Additionally, accumulated 2-HG is actively imported by the scarce infiltrating effector T cells present. In the lymphocyte cytoplasm, 2-HG disrupts mitochondrial bioenergetic coupling and alters the nuclear translocation of the NFAT factor, directly suppressing cell proliferation, effector cytokine secretion, and cytotoxic activity [62]. The net result of this epigenetic and metabolic repression is a state of profound immunological quiescence that favors slow tumor growth and extended patient survival compared to hyperinflammatory IDH-wildtype glioblastomas [16,62] (Figure 4).

### 6.2. Brain Metastases and Primary CNS Tumors

The neuroinflammatory microenvironment of secondary brain metastases (BrMs) originating from extracranial solid tumors (such as lung carcinomas, breast cancer, and melanomas) presents profound pathophysiological divergences compared to primary gliomas of astrocytic or glial lineage [21]. These differences lie both in the original tumor cell lineage and in the dynamics of neovascularization and BBB disruption. Whereas primary gliomas such as GBM diffusely infiltrate the brain tissue, partially preserving the neural ultrastructure and coexisting in an integrated manner with resident microglia and heterogeneously distributed BMDMs, metastases of epithelial or melanocytic origin initially establish themselves as expansive globular masses that cause marked local disruption of the BBB at capillary junctions [21].

Consequently, the innate immune microenvironment of BrMs is dominated almost exclusively by peripherally recruited BMDMs, which infiltrate the parenchyma of the metastatic mass. Resident parenchymal microglia are mechanically displaced toward the outer edges of the nodular mass, forming a dense peripheral defensive myeloid capsule. This capsular microglia, upon contacting the metastatic tumor metalloproteinases, up-regulates ECM remodeling genes such as osteopontin (SPP1) and GPNMB, structuring a fibrous network that can restrict the spatial distribution and efficiency of effector T lymphocytes, although active, heterogeneous lymphocyte infiltration still occurs within the metastatic stroma [21,63].

### 6.3. Meningiomas and Medulloblastomas

Outside of gliomas and secondary metastatic seeding, the CNS harbors neoplasms with specific neuroinflammatory characteristics determined by their anatomical niche. Meningiomas, originating from arachnoid cap cells, are located extra-axially, adjacent to the dural venous sinuses and outside the protection of the classical BBB parenchyma. In high-grade WHO meningiomas (grades 2 and 3), the inflammatory stroma is characterized by a dense infiltration of macrophages originating from the general circulation. A defining pathophysiological trait of neuroinflammation in aggressive meningiomas is the recurrent presence of tertiary lymphoid structures (TLS) in the dural periphery, which operate as organized local centers for antigen presentation, robustly correlating with effective antitumor clearance and increased patient survival, a finding not replicated in intraaxial neoplasms [60].

In the pediatric population, medulloblastoma is a highly aggressive embryonal tumor originating in the cerebellar posterior fossa, molecularly classified into four subgroups (WNT, SHH, Group 3, and Group 4) endowed with disparate immune profiles [60].

The WNT subgroup, characterized by an excellent therapeutic response, possesses a richly inflamed microenvironment, abundant peritumor TLS, and massive infiltration of non-exhausted effector CD8+ T lymphocytes, facilitated by fenestrated vasculature devoid of a barrier [60].

The SHH and Group 3 subgroups show a tumor parenchyma dominated by dense networks of TAMs polarized toward suppressor phenotypes and granulocytic MDSCs. These myeloid cells are reprogrammed by the signals of the developing embryonic cerebellum, actively inducing immunological tolerance and forcing lymphoid exclusion through posterior fossa cytokines, which disables the transfer of effective immunological therapies validated in adult gliomas to pediatric patients [60] (Figure 4).

## 7. Biomarkers and Diagnostic Correlates

### 7.1. BBB and BTB Disruption

The cerebral BBB, constituted by non-fenestrated endothelial cells tightly joined by claudin-5 and occludin protein complexes, sustained by an intact basement membrane and perivascularly surrounded by astrocytic podocytes and pericytes, is progressively disrupted by the proinflammatory and proangiogenic soluble factors secreted by the tumor [5]. As the glioma advances, the hypoxia of the perinecrotic niches constitutively stabilizes HIF-1α, which constitutively induces the massive transcription and secretion of VEGF-A [19,20].

Free VEGF-A couples to its cognate receptor VEGFR2 expressed on the luminal membrane of adjacent capillary endothelial cells, inducing the phosphorylation of tight junction proteins and causing their physical disassembly and subsequent internalization by endocytosis [5]. This endothelial disruption is exacerbated by the release of MMP-2 and MMP-9 metalloproteinases by perivascular TAMs, which catalytically digest type IV collagen and basal components of the neurovascular unit [5].

The consequence of this BBB disruption is the establishment of a structurally aberrant and permeable blood-tumor barrier (BTB), which allows the extravasation of water, electrolytes, and plasma immunoglobulins into the brain interstitial space, causing massive vasogenic edema. The concomitant increase in interstitial fluid pressure mechanically compresses the adjacent microvasculature, exacerbating local tissue hypoxia and generating a pro-tumor feedback loop [5]. To counteract this edema, the synthetic corticosteroid dexamethasone is widely prescribed, which stabilizes the transcription of binding proteins and reduces VEGF-A secretion. However, dexamethasone induces profound systemic and intratumor immunosuppression, blocking effector lymphocyte activation and directly sabotaging the therapeutic efficacy of agents directed against immune checkpoints [10].

### 7.2. Imaging of Pseudoprogression

The inability of conventional gadolinium contrast-enhanced MRI to reliably differentiate between true tumor progression, treatment-induced pseudoprogression, and radiation necrosis represents a methodological clinical challenge [64]. Radiotherapy and chemotherapy with temozolomide damage endothelial and tumor DNA, triggering cellular senescence and the massive release of DAMPs into the brain stroma. This signaling acutely activates the canonical NF-κB pathway in surrounding astrocytes and TAMs, causing an increase in local secretion of IL-1β, TNF-α, and VEGF-A, and triggering a transient BBB disruption that mimics active tumor growth on MRI through increased enhancement volume on T1-weighted gadolinium sequences [64].

To resolve this diagnostic conflict, advanced functional and molecular diagnostic approaches are employed. Proton Magnetic Resonance Spectroscopy (1H-MRS) allows for the quantification of the relative concentrations of key metabolites. In regions of active tumor progression, a marked elevation of the choline peak (a marker of tumor cell membrane turnover) is documented together with a depletion of N-acetylaspartate (NAA, a marker of brain neuronal integrity); conversely, tissues experiencing pseudoprogression or radiation necrosis exhibit low to moderate choline peaks accompanied by a prominent increase in the spectral signal of myo-inositol, a specific marker metabolite of reactive non-tumor glial proliferation, as well as lactate/lipid peaks derived from tissue liquefaction [64].

**Figure 5:**
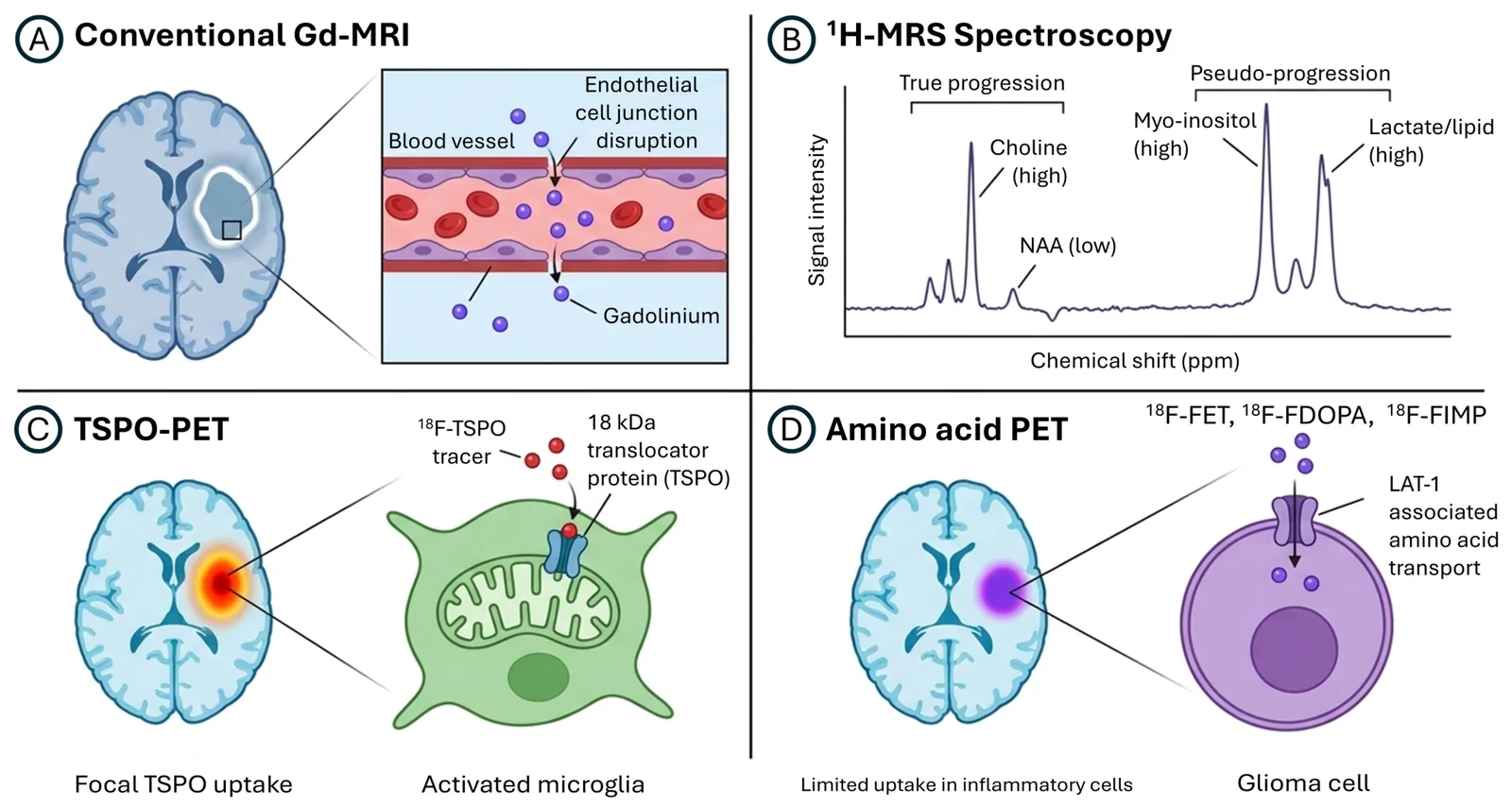
Diagnostic imaging correlates of neuroinflammation and tumor progression. (**A**) Conventional gadolinium-enhanced MRI showing perilesional enhancement. (**B**) 1H-MRS spectroscopy with choline, NAA, myo-inositol, and lactate/lipid peaks differentiating progression from pseudoprogression. (**C**) TSPO-PET uptake in activated microglia. (**D**) Amino acid PET using representative LAT1-associated tracers (^18^F-FET, ^18^F-FDOPA, and ^18^F-FIMP), showing predominantly increased uptake in glioma tissue.

On the other hand, dynamic susceptibility contrast MRI perfusion imaging (DSC-MRI) allows for the derivation of the relative cerebral blood volume (rCBV). Foci of hypervascularized tumor recurrence systematically exhibit a marked increase in rCBV due to the continuous recruitment of newly formed capillaries by tumor VEGF-A, whereas areas of neuroinflammatory pseudoprogression and radiation necrosis typically show a depletion of rCBV due to radiation-induced capillary damage and thrombosis, allowing their differential diagnostic delimitation [64] (Figure 5).

### 7.3. PET Imaging of TSPO and Other Targets

In vivo quantification of neuroinflammation has advanced through the use of [^18^F]F-DED PET, which specifically monitors reactive astrogliosis via MAO-B expression, offering a more sensitive read-out of early inflammatory activity than traditional TSPO ligands [65,66]. Furthermore, integrated radiomics-based multivariable modelling can now delineate systemic immune states from MRI patterns, accurately predicting peripheral blood T-cell populations and Th1-polarizing transcription factors like T-bet, thereby serving as a non-invasive surrogate for patient immunophenotyping [67]. The most widespread analytical target is the 18 kDa mitochondrial translocator protein (TSPO), whose expression increases in activated microglia, macrophages, and reactive astrocytes, as well as intrinsically in certain glioma strains [44].

The prognostic utility of TSPO-PET extends to IDH-mutant gliomas, where high SUVmax and PET-positive tumor volume independently predict shorter time to next intervention and overall survival [68]. Furthermore, microglial activation visualized by TSPO ligands such as [^18^F]-DPA714 has been validated as a marker of severity; in inflammatory mimics like LGI1 encephalitis, DPA714 uptake correlates significantly with sTREM2 levels in the CSF and cognitive impairment [44]. To visualize the transition toward an anti-inflammatory phenotype, the P2Y12-targeting tracer [^18^F]QTFT offers a diagnostic tool, showing high uptake in restorative microglial states during tumor progression and injury resolution [18]. Patients exhibiting a high TSPO-PET signal (>1.8 mean SUV) have a shortened overall survival of 8.3 months compared to 17.8 months for those with low baseline uptake, operating as an independent negative prognostic factor [68]. Additionally, TSPO PET has demonstrated its ability to differentiate true progression from pseudoprogression by capturing the enhancement of the reparative immune infiltrate of radiation [44]. However, the human genetic polymorphism rs6971 in the TSPO gene substantially alters the binding affinity of second-generation radioligands such as 11C-PBR28 or 18F-GE180, classifying patients as high, medium, or low affinity binders, and detracting from absolute diagnostic reproducibility unless prior genotyping is performed [68] (Figure 5).

To circumvent these constraints, PET directed against TREM2 is under active investigation, a surface receptor expressed exclusively in activated parenchymal microglia and suppressed in glioma tumor cells and astrocytes. Developed TREM2 radioligands (such as conjugates of the 14D3 microglia antibody) allow specific and high-resolution capture of the density of the reactive myeloid infiltrate surrounding the tumor, without interference from the mitochondrial signal of the tumor cells, operating as a dynamic real-time monitor of the adaptive immune response following the administration of cell re-education therapies or CAR-T cell infusion [44].

In parallel, to circumvent the limitations of 18F-FDG PET, which undergoes high non-specific uptake in glycolytic, inflammatory macrophages, amino acid PET imaging targeting system L transporters (LAT1/2) has been increasingly integrated into clinical practice. Tracers such as O-(2-[^18^F]fluoroethyl)-L-tyrosine ([^18^F]FET), 3,4-dihydroxy-6-[^18^F]-fluoro-L-phenylalanine ([^18^F]FDOPA), and [^18^F]FIMP target amino acid transport systems, including LAT1, and generally show increased uptake in glioma tissue compared with non-neoplastic brain. Their relatively low uptake in inflammatory tissue may provide complementary information for distinguishing tumor progression from treatment-related inflammatory changes, although amino acid PET should not be regarded as a specific marker of neuroinflammation. This uptake pattern may therefore provide complementary information for distinguishing active tumor progression from treatment-related inflammatory changes, although it does not provide specific imaging of neuroinflammation [68,69]. Radiomic imaging now allows for the delineation of systemic immune activity states, such as the blood abundance of T cell populations or specific transcription factors, by correlating MRI patterns in the necrotic core and peritumoral zone [67]. Complementarily, multimodal mapping has validated that imaging-detected microvascular pathology and vasogenic edema are robust predictors of failure in neoadjuvant immunotherapy [10] (Table 2). To complement neuroimaging, the emerging paradigm of ‘glial liquid biopsy’ from cerebrospinal fluid (CSF) or peripheral blood offers a promising non-invasive tool to monitor neuroinflammation. This approach isolates tumor- and astrocyte-derived extracellular vesicles (EVs), circulating cell-free nucleic acids, and cytokine profiles to capture real-time, longitudinal shifts in the inflammatory microenvironment during treatment [10,33].

## 8. Therapeutic Strategies and Translational Challenges

### 8.1. CSF1R Inhibition

The role of TAMs in propagating immunological tolerance and tissue remodeling motivated the exploration of the CSF1-CSF1R pathway as a primary therapeutic target [71,72]. Small CNS-penetrating molecules inhibiting CSF1R tyrosine kinase activity, such as pexidartinib (PLX3397) or BLZ945, demonstrated outstanding antitumor efficacy in preclinical rodent models, inducing the regression of established brain tumors and improving survival curves [71]. Histological analyses revealed that therapeutic efficacy did not reside in inducing widespread myeloid apoptosis but in forcing a profound reprogramming or re-education of the surviving TAMs, which decreased their immunosuppressive genes and recovered their phagocytic and cytotoxic antigen-presenting capacity [71].

However, subsequent phase II clinical trials in patients with recurrent human glioblastoma yielded negative results, with no statistically significant differences in progression-free survival or overall survival [73]. This clinical translatability gap is a consequence of the activation of redundant compensatory mechanisms in the human TME [5]. Persistent blockade of CSF1R signaling induces tumor cells to secrete elevated amounts of insulin-like growth factor 1 (IGF-1). Free IGF-1 binds to its transmembrane tyrosine kinase receptor IGF-1R, whose expression is compensatorily upregulated on the surface of the surviving TAMs post-depletion. This binding stimulates the PI3K/Akt mitogenic pathway in parallel and independently of CSF1R signaling, forcing the suppressor repolarization and maintenance of intratumor macrophages [5].

Simultaneously, local depletion of macrophages activates the release of granulocyte-recruiting chemokines by the glioma, promoting a massive compensatory infiltration of PMN-MDSCs that immediately restore the shield of intratumor immunological tolerance, nullifying the clinical benefit of the drug [5]. Furthermore, long-term studies reveal that approximately 50% of recurrences following anti-CSF1R therapy are associated with the formation of fibrotic scars. These fibrotic niches, mediated by perivascular fibroblast-like cells via TGF-β signaling and neuroinflammation, encapsulate surviving glioma cells and promote dormancy and immune evasion [72].

In addition to small molecule inhibitors, the natural isoflavonoid calycosin has shown promise in modulating these pathways. Calycosin suppresses the HMGB1/TLR4/NF-κB axis and inhibits glioblastoma migration by downregulating TGF-β-mediated mesenchymal properties and the c-Met/CXCL10 pathways [74,75]. Furthermore, calycosin synergizes with temozolomide by activating caspase-3/9 mediated apoptosis, offering a potential adjunctive strategy to overcome the resistance seen with single-agent CSF1R blockade [74,75].

### 8.2. Innate Immune Checkpoint Blockade

To restore the innate myeloid capacity for tumor phagocytosis, blocking antibodies against surface protective or “don’t eat me” signals have been developed [76]. The classic target is the glycoprotein CD47, overexpressed on the glioma membrane and bound to the myeloid receptor SIRPα, triggering the phosphorylation of intracellular ITIM domains and inhibiting the actin polymerization of the phagocytic cup. Antibodies such as magrolimab have shown robust preclinical phagocytosis induction, but their clinical translation is hindered by collateral hemolytic anemia due to basal CD47 expression on senescent erythrocytes [76].

To circumvent this systemic limitation and increase the specificity of innate activation, leukocyte immunoglobulin-like receptor subfamily B member 1 (LILRB1, or ILT2) has emerged as a high-impact target. LILRB1 is an inhibitory receptor abundantly expressed on TAMs, dendritic cells, NK cells, and T cell subpopulations [33]. This receptor directly interacts with classical and non-classical MHC class I components (such as HLA-G) expressed on tumor cells, transmitting negative cytoplasmic signals through its ITIM domains [33].

Molecular studies confirm that LILRB1 expression is drastically increased in high-grade WHO gliomas, independently correlating with a depressed clinical prognosis [33]. Gene set enrichment analysis (GSEA) has revealed that LILRB1 expression closely correlates with the transcriptional activation of the JAK/STAT3 signaling pathway [33]. Pathophysiologically, glioma cells constitutively induce the up-regulation of the β2-microglobulin protein (β2m), which stabilizes surface MHC-I and potentiates LILRB1 binding on surrounding macrophages, educating these cells toward an M2-type suppressor phenotype [33]. The dual selective blockade of the CD47-SIRPα and LILRB1-MHC-I axes using monoclonal antibodies with Fc-silent modification (to abrogate erythrocytic toxicity) synergistically restores productive myeloid phagocytosis of tumor cells, unifying innate activation against clones that down-regulate MHC-I to evade CD8+ adaptive lymphocyte action [33].

### 8.3. Cellular Immunotherapy

The incorporation of T lymphocytes modified to express chimeric antigen receptors (CAR-T) directed against single targets in glioblastoma, such as EGFRvIII, IL-13Rα2, or HER2, has historically stumbled upon the heterogeneity of tumor antigens and the adaptive immune paralysis mediated by the TME [77,78]. Following the administration of CAR-T directed against EGFRvIII, a clonal depletion of positive cells was observed, but recurrence was dominated by mutated cell clones lacking the expression of said antigen [77,78].

To synergistically neutralize this antigen escape, the development of next-generation cellular products has been implemented, highlighting the bivalent cellular construct CARv3-TEAM-E, evaluated in the phase I clinical trial INCIPIENT (NCT05660369) [77]. These autologous T cells are genetically modified to express a second-generation CAR receptor with CD3ζ and 4-1BB activation domains directed specifically against the truncated neoantigen EGFRvIII. However, the innovation of this construct lies in the fact that it contains a secretion cassette for T cell-engaging antibody molecules (TEAMs) of dual specificity directed against the wildtype EGFR receptor (anti-CD3/anti-EGFR) [77,78].

This molecular design confers on the modified T cell a dual cytotoxic activation pathway: it directly eliminates EGFRvIII+ tumor cells through its CAR domain, while locally secreting the bispecific TEAM molecules into the tumor parenchyma. The secreted TEAMs bind to native EGFR present on the surface of adjacent tumor cells and recruit unmodified polyclonal native T lymphocytes of the host that infiltrate the lesion, overcoming the barrier of antigenic escape and clearing heterogeneous tumor clones [77,78]. Phase I clinical evaluations revealed that rapid radiological responses of tumor mass decrease within days of a single local infusion administered intraventricularly via an Ommaya reservoir. However, in two of the three initial patients these regressions were transient, associated with limited CAR-T cell persistence [77,78]. To mitigate the associated neurotoxicity and cognitive impairment of combined chemoradiotherapy, human neural stem cell-derived extracellular vesicles (hNSC-EVs) are emerging as a translational strategy; these vesicles preserve synaptic integrity and improve memory performance without interfering with antitumor efficacy [79].

Subsequent analyses of this clinical trial determined that the loss of immune persistence was a consequence of the immunogenicity of the human–murine recombinant construct, detecting the biosynthesis of anti-CAR and anti-TEAM IgG antibodies in the CSF in 9 out of 10 patients who received the product in isolation, thereby nullifying the efficacy of the scheduled subsequent infusions [77]. To overcome this translational immunological impediment, a critical modification was introduced in the patient preconditioning protocol. The addition of the B cell-depleting monoclonal antibody rituximab to the standard lymphodepleting chemotherapy regimen with fludarabine and cyclophosphamide (LDC + R) prevented the generation of therapy-blocking humoral antibodies, significantly prolonging the persistence and viability of active CARv3-TEAM-E cells in the CSF of all patients undergoing successive reinfusions [77].

### 8.4. Recent Clinical Trials

Translational research in neuro-oncology for the 2025/2026 period is defined by the methodological transition toward the simultaneous inhibition of concurrent tumor communication networks with the nervous and immune systems, highlighting three main milestones:

GIANT Clinical Trial (NCT06816927): This multicenter, randomized phase II study investigates the feasibility, tolerance, and pharmacodynamic biomarkers of neoadjuvant immunotherapy combining nivolumab (anti-PD-1) with relatlimab, a monoclonal antibody designed to block the LAG-3 immune checkpoint molecule expressed on exhausted TILs, in patients with newly diagnosed IDH-wildtype glioblastoma [10]. The molecular hypothesis posits that the administration of a combined initial neoadjuvant dose before surgical tumor resection, leveraging the presence of the intact tumor mass as a myeloid cellular antigen stimulator, induces a superior clonal expansion and priming of effector T lymphocytes compared to isolated post-surgical adjuvant regimens [10]. After surgery, the administration of the combined treatment is resumed, associated with the standard radiotherapy and chemotherapy regimen [10].

SENIPERA Trial Platform (NCT07284069/EU-CT 2025-522605-37-00): It represents the first direct clinical translation of discoveries in cancer neuroscience to the human clinic [12,80]. The SENIPERA-1 trial (phase 0/1) evaluates the safety, pharmacokinetics, and tissue penetration of a dual combinatorial strategy directed against tumor communication networks. It uses perampanel, a non-competitive AMPA-type glutamate receptor antagonist approved for epilepsy, to selectively interrupt the excitatory synaptic transmission that fuels tumor proliferation [80]. Simultaneously, it introduces senicapoc, a small selective blocking molecule of intermediate-conductance calcium-activated potassium channels (IKCa), responsible for regulating the cytosolic calcium oscillations of the tumor syncytial network [80]. Based on the tolerance shown in SENIPERA-1, in 2026 the development of SENIPERA-2 was funded, proposed as a multicenter, randomized, double-blind phase II clinical trial aimed at demonstrating whether the addition of perampanel and senicapoc to the standard chemoradiotherapy regimen significantly improves patient progression-free survival [80].

Modeyso Approval (Dordaviprone/ONC201): On 6 August 2025, the FDA granted accelerated approval to dordaviprone (Modeyso, Jazz Pharmaceuticals) for the treatment of adult and pediatric patients with diffuse midline glioma (DMG) harboring the H3 K27M mutation and presenting disease progression after radiotherapy [60]. The H3 K27M histone mutation causes a deleterious epigenetic remodeling characterized by the depletion of H3K27me3 methylation [60]. Pathophysiologically, dordaviprone is a small oral molecule that acts as a selective allosteric agonist of the mitochondrial protease ClpP, inducing its uncontrolled activation and the subsequent enzymatic degradation of critical proteins of the electron transport chain, collapsing tumor cell metabolism, while competitively antagonizing the D2 dopamine receptors (DRD2) overexpressed in these tumors, marking the first approval milestone of a targeted systemic therapy against this pathology [60].

Differential diagnosis remains a critical hurdle; high-grade gliomas can uncommonly present with an autoimmune encephalitis (AIE)-like clinical picture, including seizures and CSF pleocytosis. Clinicians must apply strict criteria, as anti-neuronal antibody positivity (e.g., NMDAR-IgG) in patients with infiltrative, FDG-avid lesions can be misleading, delaying the definitive diagnosis of IDH-wildtype glioblastoma [81,82].

**Figure 6:**
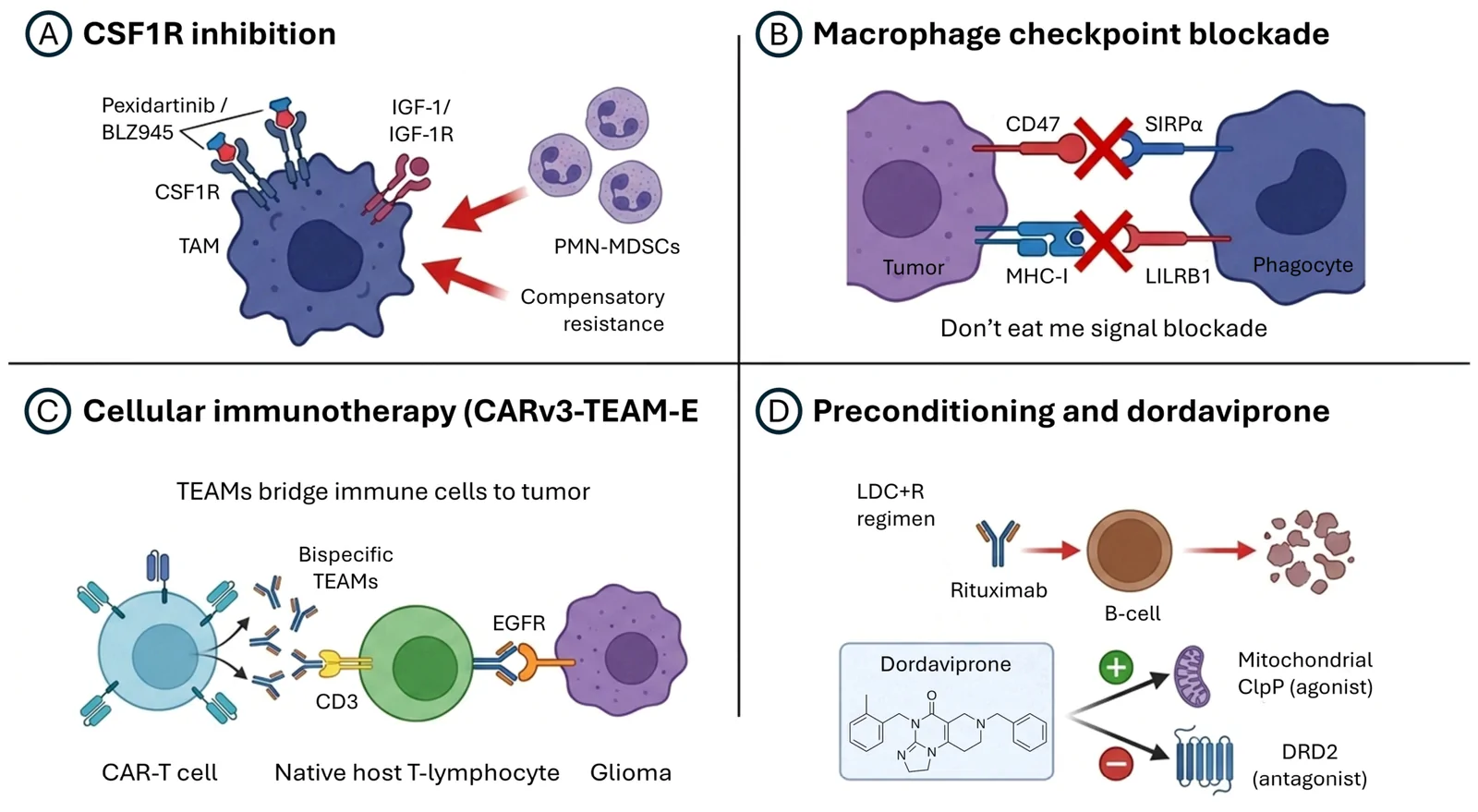
Current and emerging therapeutic strategies targeting neuroinflammation and tumor-immune interactions. (**A**) CSF1R inhibition (pexidartinib, BLZ945) with compensatory resistance mechanisms (IGF-1/IGF-1R, PMN-MDSC recruitment). (**B**) Blockade of “don’t eat me” signals (CD47-SIRPα and LILRB1-MHC-I). (**C**) CARv3-TEAM-E cellular immunotherapy: CAR-T cell secreting bispecific TEAMs (anti-CD3/anti-EGFR) recruiting host polyclonal T lymphocytes. (**D**) LDC + R preconditioning regimen with rituximab to prevent immunogenicity. Inset: Dordaviprone mechanism (mitochondrial ClpP agonist and DRD2 antagonist).

Recent clinical trials targeting neuroinflammation and neuron–glioma signaling pathways, including their mechanisms, phases, and main translational features, are summarized in Table 3 (Figure 6).

## 9. Discussion and Clinical Perspectives

Despite the expansion in the dissection of the molecular and immunometabolic neuroinflammatory mechanisms described here, critical conceptual and methodological limitations persist that slow their direct therapeutic applicability in patients with CNS neoplasms.

Firstly, the incomplete fidelity of animal models: Most mechanistic data on recruitment and cytokine transduction come from implanted orthotopic syngeneic rodent lines (such as GL261 or CT2A) or genetically engineered murine models (GEMMs). Although they retain a functional immune system, it has been shown that the transcriptomic profiles and the composition of mouse myeloid and glial receptors differ substantially from their human counterparts in situ [4,21]. Conversely, patient-derived xenograft (PDX) models require the use of immunocompromised hosts devoid of functional adaptive cellular immunity, which limits rigorous study of lymphocyte exhaustion and reciprocal myeloid-lymphoid communication [4,21,83]. To overcome these drawbacks, future translational research must utilize humanized mouse models. Reconstituting immunodeficient mice with human CD34+ hematopoietic stem cells or peripheral blood mononuclear cells establishes a functional human immune system in vivo, providing an essential platform to investigate human-specific stroma-immune interactions and evaluate emerging immunotherapies under physiological conditions [83].

Secondly, the static nature of clinical analyses: A large part of the findings in humans are derived from the retrospective analysis of biopsies and surgical samples taken at a single specific time point of the disease. Brain neuroinflammation is a highly dynamic and changing phenomenon that substantially evolves under the pressure of radio and chemotherapy, selecting cell clones endowed with disparate adaptive escape mechanisms that cannot be captured by conventional static longitudinal approaches [5,84].

**Figure 7:**
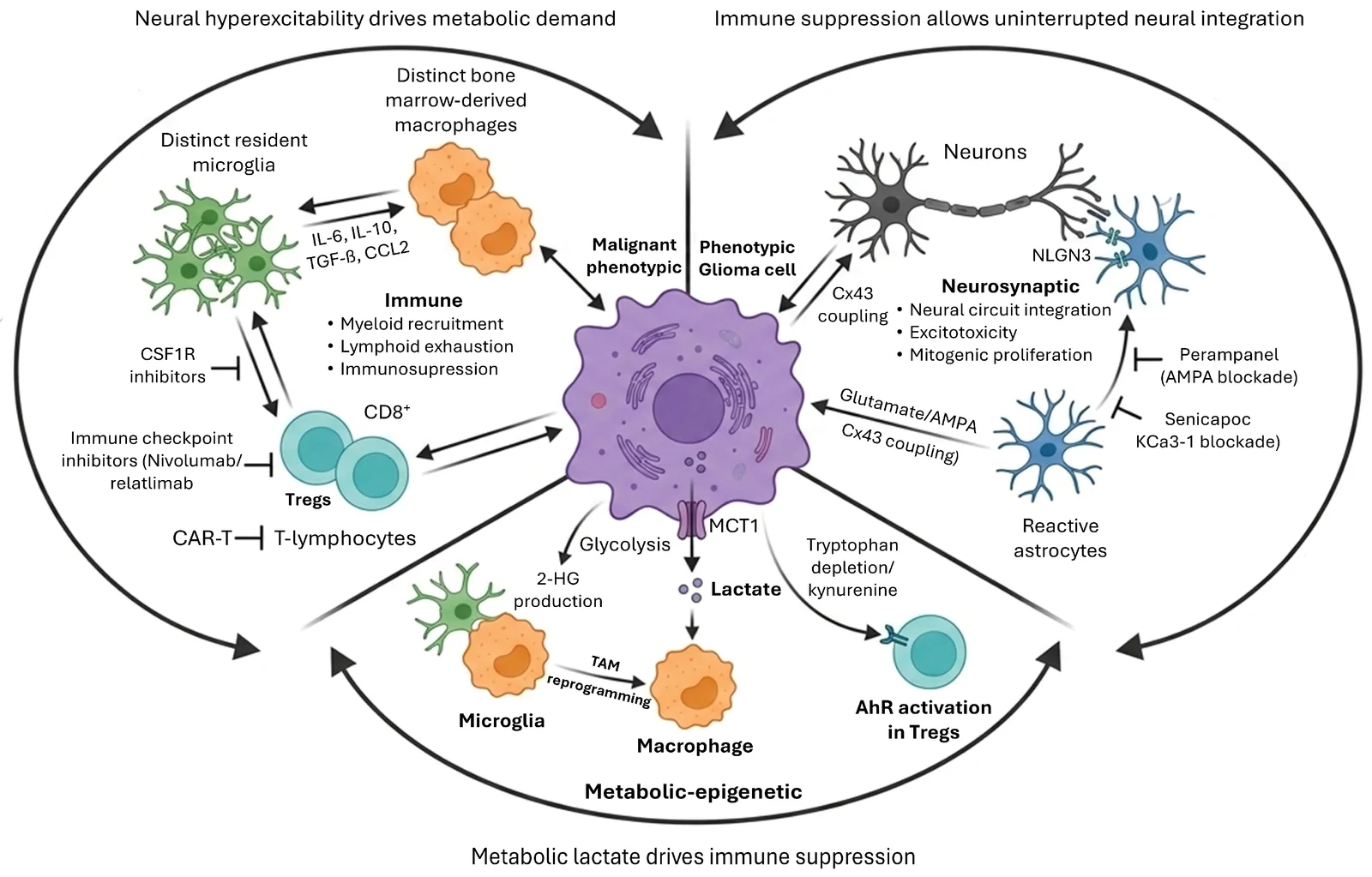
Integrated model of neuroinflammation pathophysiology in glioblastoma. The three main axes are connected: (1) immune axis (myeloid recruitment, lymphoid exhaustion, immunosuppression); (2) metabolic-epigenetic axis (glycolysis, lactate, 2-HG, TAM reprogramming); (3) neurosynaptic axis (neural circuit integration, excitotoxicity, mitogenic proliferation). Interconnections between axes and therapeutic intervention points (CSF1R, checkpoints, perampanel/senicapoc, CAR-T) are indicated.

Third, the pharmacological shield of corticosteroids: The chronic symptomatic management of intracranial hypertension using dexamethasone profoundly alters the intratumor immune and myeloid profile of patients analyzed in clinical trials. Dexamethasone suppresses lymphocyte chemotaxis and abolishes the transactivation of effector immune genes [70,85]. Developing and implementing unified clinical steroid-sparing protocols, incorporating early the edema-blocking molecules that do not compromise the cellular immune response (such as losartan through suppression of astrocytic TGF-β synthesis, or bevacizumab in a controlled manner), is a high methodological priority requirement to calibrate the real efficacy of new immunotherapeutic agents in the clinic [70,85] (Figure 7).

The clinical future lies at the intersection of cancer neuroscience, TME metabolism, and cellular immunology. The incorporation of human brain organoids co-cultured in a controlled manner with microglia, astrocytes, and autologous lymphocytes, together with the analysis of myeloid transcriptomes from serial liquid biopsies derived from the CSF, promises to refine the predictive specificity of molecular targets before their implementation in humans. Only through approaches that integrally address cerebral neuroinflammatory functional redundancy, stromal cellular plasticity, and tumor bioelectric coupling with the neuronal network will it be possible to reverse the biological and therapeutic progression of these lethal intracranial neoplasms.

## Figures and Tables

**Table 1:** Cellular components of the tumor immune microenvironment (TIME) in the CNS.

Cell Type	Ontogeny	Identification Markers	Pathophysiological Function in the TIME	Refs.
Resident microglia	Yolk sac (embryonic)	TMEM119+, P2RY12^+^, CX3CR1^+^, CD45^low^	Peripheral surveillance; matrix remodeling at infiltration margins.	[15,16]
Recruited BMDMs	Bone marrow (monocytes)	CD45^high^, CD49d^+^, CD163^+^	Tumor core and necrotic area infiltration; profound immunosuppression and angiogenesis.	[16,21]
Reactive astrocytes	Local parenchyma	GFAP^+^, Vimentin^+^, Cx43^+^	Glial scar formation; tumor metabolic protection and physical lymphocyte exclusion.	[13,33]
PMN-MDSCs	Bone marrow	CD11b^+^CD14^−^CD15^+^	L-Arginine depletion (ARG-1); ROS production; induction of T cell anergy.	[34,35]
M-MDSCs	Bone marrow	CD11b^+^CD14^+^HLA-DR^−/low^	iNOS production; T cell suppression via arginine and nitric oxide depletion.	[34,35]
Exhausted CD8+ T lymphocytes	Thymus (periphery)	PD-1^+^CTLA-4^+^TIM-3^+^LAG-3^+^	Functional exhaustion; cytotoxic incapacity; reduced production of IFN-γ and TNF-α.	[36]
Regulatory T lymphocytes (Tregs)	Thymus (periphery)	CD4^+^CD25^+^FoxP3^+^CCR4^+^	Suppression of effector immunity; IL-10 and TGF-β secretion; adenosine production.	[37,38]

**Table 2:** Molecular pathways in neuroinflammation: Pathophysiological roles and experimental methodologies.

Molecule/Pathway	Physiological Role	Pathological Subversion in CNS Tumors	Experimental Methodologies	Refs.
cGAS-STING pathway	Intracellular sensor of cytosolic double-stranded DNA (dsDNA) from viral infection or cellular damage; induces type I interferons.	Epigenetically silenced in glioma cells to evade immune recognition; subverted by channeling tumor cGAMP to astrocytes via Cx43 gap junctions.	Western blotting, qRT-PCR, cyclic dinucleotide ELISA, STING-reporter assays, immunofluorescence for micronuclei.	[49,50]
NLRP3 inflammasome	Multiprotein cytosolic complex that senses cellular danger-associated molecular patterns (DAMPs); activates caspase-1.	Chronically activated in TAMs in response to necrotic tumor ATP and ROS, leading to sustained pro-tumorigenic IL-1β secretion.	Caspase-1 activity assays, ELISA for mature IL-1β, immunofluorescence for ASC specks, Western blot.	[41,47]
STAT3 signaling	Latent cytoplasmic transcription factor activated by cytokines (IL-6, IL-10); regulates cell growth and survival.	Constitutively phosphorylated (pY705) in tumor cells and TAMs, directing immunosuppressive and angiogenic gene transcription.	Flow cytometry (phospho-STAT3 staining), chromatin immunoprecipitation (ChIP-seq), Western blot, luciferase reporter assays.	[16,46]
NF-κB pathway	Master regulator of inflammation, cell survival, and stress responses downstream of TLRs and cytokine receptors.	Constitutively active in mesenchymal gliomas, driving the transcription of anti-apoptotic (Bcl-xL) and pro-tumor cytokine (IL-6, IL-8) genes.	Western blot for IκBα degradation and p65 nuclear translocation, Electrophoretic Mobility Shift Assay (EMSA), ChIP-seq.	[47,70]
Aquaporin-4 (AQP4)	Astrocytic water channel localized to perivascular endfeet; maintains brain fluid homeostasis and glymphatic clearance.	Loss of perivascular polarization (depolarization), leading to disrupted glymphatic solute clearance, edema, and neural desynchronization.	High-resolution immunofluorescence (polarity index calculation), glymphatic tracer imaging (contrast-enhanced MRI), Western blot.	[2,28]
AMPA receptors (GluA1–4)	Ionotropic glutamate receptors mediating fast, excitatory synaptic transmission in neuronal networks.	Expressed on tumor microtubes (TMs), forming functional synapses with presynaptic neurons that bioelectrically fuel glioma proliferation.	Whole-cell patch-clamp electrophysiology, live-cell calcium imaging, immunohistochemistry, electron microscopy.	[56,57]
SARM1 enzyme	Pro-degenerative NAD+ hydrolase that functions as the central executioner of Wallerian degeneration following axonal injury.	Activated by tumor expansion into white matter, triggering Wallerian degeneration of axons and driving pro-tumorigenic neuroinflammation.	Western blot, mass spectrometry (NAD+ depletion assays), immunohistochemistry for neurofilaments and synaptic puncta.	[11]
C5a/C5aR1 axis	Complement anaphylatoxin and its receptor; mediate leukocyte chemotaxis and proinflammatory signaling.	Facilitates glioma stem cell-TAM symbiosis, driving TAM recruitment via p-AKT and protecting tumor cells from CD8+ T cell clearance.	Flow cytometry for C5aR1, chemotaxis assays (Transwell), in vivo pharmacology (using C5aR1 antagonist PMX205).	[39,40]
TREM2/TIM3 axis	Scavenger and checkpoint receptors regulating microglial homeostasis, survival, and peripheral macrophage suppression.	Drastically upregulated during chronological brain aging, driving the extravasation of suppressive TAMs that promote immune evasion.	Single-cell/Single-nucleus RNA-seq (scRNA-seq/snRNA-seq), high-dimensional flow cytometry/CyTOF, immunohistochemistry.	[17]
NKCC1/KCC2 cotransporters	Chloride cotransporters regulating intracellular chloride concentration and GABAergic hyperpolarization.	NKCC1 overexpression and KCC2 deficiency in DMG cells depolarize the chloride gradient, converting GABA into a proliferative engine.	Short-circuit current measurements, chloride-sensitive dye imaging (e.g., MQAE), Western blot, patch-clamp recordings.	[56,58]

**Table 3:** Clinical Trials in Neuroinflammation and Neural Networks.

Trial/Identifier	Drug(s) Tested	Pathophysiological Mechanism/Target	Clinical Phase and Population	Highlighted Findings/Novelty	Refs.
GIANT (NCT06816927)	Nivolumab + Relatlimab (neoadjuvant)	PD-1 blockade + LAG-3 blockade in a perioperative regimen	Phase II, newly diagnosed IDH-wildtype GBM	Neoadjuvant regimen aims to maximize peripheral T lymphocyte priming before surgical resection.	[10]
SENIPERA-2 (Denmark)	Perampanel + Senicapoc	AMPA blockade (neuro-tumor synapse) + KCa3.1 blockade (TMs)	Phase II, newly diagnosed GBM	Funded by the Lundbeck Foundation in 2026 to evaluate impact on progression-free survival.	[12,80]
INCIPIENT update (CARv3-TEAM-E)	CARv3-TEAM-E + LDC + R	CAR-T against EGFRvIII/EGFR + Rituximab to prevent immunogenicity	Phase I, recurrent GBM	Rituximab eradicated the formation of antibodies against therapy and safely prolonged cellular persistence in CSF.	[77]
Modeyso™ (Jazz Pharmaceuticals)	Dordaviprone (ONC201)	Mitochondrial ClpP activation + DRD2 antagonism	Accelerated approval (August 2025), DMG H3 K27M	First targeted therapy approved for diffuse midline gliomas with H3 K27M histone mutation.	[60]

## Data Availability

No new data were created or analyzed in this study. Data sharing is not applicable to this article.

## Abbreviations

The following abbreviations are used in this manuscript:

Abbreviation	Meaning
1H-MRS	Proton magnetic resonance spectroscopy
2-HG	D-2-hydroxyglutarate
AhR	Aryl hydrocarbon receptor
AKT	Protein kinase B
AMPA	Alpha-amino-3-hydroxy-5-methyl-4-isoxazolepropionic acid
AQP4	Aquaporin-4
ARNT	Aryl hydrocarbon receptor nuclear translocator
ASC	Apoptosis-associated speck-like protein containing a CARD
ATP	Adenosine triphosphate
BBB	Blood–brain barrier
BDNF	Brain-derived neurotrophic factor
BMDMs	Bone marrow-derived macrophages
BrMs	Brain metastases
BTB	Blood-tumor barrier
bFGF	Basic fibroblast growth factor
cAMP	Cyclic adenosine monophosphate
cGAMP	Cyclic GMP-AMP
cGAS	Cyclic GMP-AMP synthase
CAR-T	Chimeric antigen receptor T cell
CARv3-TEAM-E	Bivalent chimeric antigen receptor T-cell platform targeting EGFRvIII/EGFR with secreted T-cell-engaging antibody molecules
CNS	Central nervous system
CSF	Cerebrospinal fluid
CSF1R	Colony-stimulating factor 1 receptor
CTLA-4	Cytotoxic T-lymphocyte-associated protein 4
Cx43	Connexin 43
DAMPs	Damage-associated molecular patterns
DMG	Diffuse midline glioma
DMGs	Diffuse midline gliomas
DNMT	DNA methyltransferase
DRD2	Dopamine receptor D2
DSC-MRI	Dynamic susceptibility contrast magnetic resonance imaging
ECM	Extracellular matrix
EGFR	Epidermal growth factor receptor
EGFRvIII	Epidermal growth factor receptor variant III
EPSCs	Excitatory postsynaptic currents
ERK	Extracellular signal-regulated kinase
EVs	Extracellular vesicles
FAO	Fatty acid oxidation
FDA	Food and Drug Administration
FOXP3	Forkhead box P3
GABA	Gamma-aminobutyric acid
GABA_A	Gamma-aminobutyric acid type A receptor
G-CIMP	Glioma CpG island methylator phenotype
GBM	Glioblastoma
GCN2	General control nonderepressible 2
GDNF	Glial cell line-derived neurotrophic factor
GEMMs	Genetically engineered mouse models
GFAP	Glial fibrillary acidic protein
GL261	Murine glioma model GL261
GPNMB	Glycoprotein nonmetastatic melanoma protein B
GSCs	Glioma stem cells
HDAC	Histone deacetylase
HER2	Human epidermal growth factor receptor 2
HIF-1α	Hypoxia-inducible factor 1-alpha
HLA-G	Human leukocyte antigen G
HPA	Hypothalamic–pituitary–adrenal
H3 K27M	Histone H3 lysine 27-to-methionine mutation
H3K4me3	Trimethylation of histone H3 lysine 4
IBA1	Ionized calcium-binding adapter molecule 1
IDH	Isocitrate dehydrogenase
IDH1/2	Isocitrate dehydrogenase 1 and 2
IFN-γ	Interferon gamma
IGF-1R	Insulin-like growth factor 1 receptor
IKK	IκB kinase
IKCa	Intermediate-conductance calcium-activated potassium channel
IL	Interleukin
iNOS	Inducible nitric oxide synthase
ITIM	Immunoreceptor tyrosine-based inhibitory motif
JAK	Janus kinase
KCC2	Potassium-chloride cotransporter 2
LAG-3	Lymphocyte activation gene 3
LAT1	L-type amino acid transporter 1
LDC + R	Lymphodepleting chemotherapy plus rituximab
LGMN	Legumain
LILRB1	Leukocyte immunoglobulin-like receptor subfamily B member 1
MAPK	Mitogen-activated protein kinase
MCT1	Monocarboxylate transporter 1
MCT4	Monocarboxylate transporter 4
MDSCs	Myeloid-derived suppressor cells
MHC	Major histocompatibility complex
MHC-I	Major histocompatibility complex class I
MHC-II	Major histocompatibility complex class II
miR	MicroRNA
MMP	Matrix metalloproteinase
MRI	Magnetic resonance imaging
mTOR	Mechanistic target of rapamycin
NAA	N-acetylaspartate
NEAT1	Nuclear enriched abundant transcript 1
NETs	Neutrophil extracellular traps
NFAT	Nuclear factor of activated T cells
NF-κB	Nuclear factor kappa B
NLGN3	Neuroligin-3
NK	Natural killer
NKCC1	Sodium-potassium-chloride cotransporter 1
NLRP3	NLR family pyrin domain-containing 3
PD-1	Programmed cell death protein 1
PD-L1	Programmed death-ligand 1
PDX	Patient-derived xenograft
PET	Positron emission tomography
PGE2	Prostaglandin E2
PI3K	Phosphoinositide 3-kinase
PMN-MDSCs	Polymorphonuclear myeloid-derived suppressor cells
PKA	Protein kinase A
P2X7R	Purinergic receptor P2X7
ROS	Reactive oxygen species
rCBV	Relative cerebral blood volume
scRNA-seq	Single-cell RNA sequencing
SARM1	Sterile alpha and TIR motif-containing protein 1
SHH	Sonic hedgehog
SHP-2	Src homology region 2-containing protein tyrosine phosphatase 2
SIRPα	Signal regulatory protein alpha
SPP1	Secreted phosphoprotein 1
STAT3	Signal transducer and activator of transcription 3
STAT6	Signal transducer and activator of transcription 6
STING	Stimulator of interferon genes
SUV	Standardized uptake value
TAAs	Tumor-associated astrocytes
TAMs	Tumor-associated macrophages
TEAM	T-cell-engaging antibody molecule
TDO2	Tryptophan 2,3-dioxygenase
TGF-β	Transforming growth factor beta
TGFBR1/2	Transforming growth factor beta receptor 1/2
TIGIT	T-cell immunoreceptor with Ig and ITIM domains
TILs	Tumor-infiltrating lymphocytes
TIM-3	T-cell immunoglobulin and mucin-domain-containing 3
TLR	Toll-like receptor
TME	Tumor microenvironment
TMs	Tumor microtubes
TNF-α	Tumor necrosis factor alpha
TSPO	18 kDa translocator protein
Tregs	Regulatory T cells
VEGF	Vascular endothelial growth factor
VEGFR2	Vascular endothelial growth factor receptor 2
VDCCs	Voltage-dependent calcium channels
WHO	World Health Organization
